# Exploring the role of professional identity in the implementation of clinical decision support systems—a narrative review

**DOI:** 10.1186/s13012-024-01339-x

**Published:** 2024-02-12

**Authors:** Sophia Ackerhans, Thomas Huynh, Carsten Kaiser, Carsten Schultz

**Affiliations:** Kiel Institute for Responsible Innovation, University of Kiel, Westring 425, 24118 Kiel, Germany

**Keywords:** Professional identity, Identity threat, Clinical decision support system, Health care, Implementation, Review

## Abstract

**Background:**

Clinical decision support systems (CDSSs) have the potential to improve quality of care, patient safety, and efficiency because of their ability to perform medical tasks in a more data-driven, evidence-based, and semi-autonomous way. However, CDSSs may also affect the professional identity of health professionals. Some professionals might experience these systems as a threat to their professional identity, as CDSSs could partially substitute clinical competencies, autonomy, or control over the care process. Other professionals may experience an empowerment of the role in the medical system. The purpose of this study is to uncover the role of professional identity in CDSS implementation and to identify core human, technological, and organizational factors that may determine the effect of CDSSs on professional identity.

**Methods:**

We conducted a systematic literature review and included peer-reviewed empirical studies from two electronic databases (PubMed, Web of Science) that reported on key factors to CDSS implementation and were published between 2010 and 2023. Our explorative, inductive thematic analysis assessed the antecedents of professional identity-related mechanisms from the perspective of different health care professionals (i.e., physicians, residents, nurse practitioners, pharmacists).

**Results:**

One hundred thirty-one qualitative, quantitative, or mixed-method studies from over 60 journals were included in this review. The thematic analysis found three dimensions of professional identity-related mechanisms that influence CDSS implementation success: perceived threat or enhancement of professional control and autonomy, perceived threat or enhancement of professional skills and expertise, and perceived loss or gain of control over patient relationships. At the technological level, the most common issues were the system’s ability to fit into existing clinical workflows and organizational structures, and its ability to meet user needs. At the organizational level, time pressure and tension, as well as internal communication and involvement of end users were most frequently reported. At the human level, individual attitudes and emotional responses, as well as familiarity with the system, most often influenced the CDSS implementation. Our results show that professional identity-related mechanisms are driven by these factors and influence CDSS implementation success. The perception of the change of professional identity is influenced by the user’s professional status and expertise and is improved over the course of implementation.

**Conclusion:**

This review highlights the need for health care managers to evaluate perceived professional identity threats to health care professionals across all implementation phases when introducing a CDSS and to consider their varying manifestations among different health care professionals. Moreover, it highlights the importance of innovation and change management approaches, such as involving health professionals in the design and implementation process to mitigate threat perceptions. We provide future areas of research for the evaluation of the professional identity construct within health care.

**Supplementary Information:**

The online version contains supplementary material available at 10.1186/s13012-024-01339-x.

Contributions to the literature

- We provide a comprehensive literature review and narrative synthesis of the role of professional identity in CDSS implementation among diverse health care professionals and identify human, technological, and organizational determinants that influence professional identity and implementation.
- The review shows that a perceived threat to professional identity plays a significant role in explaining failures of CDSS implementation. As such, our study highlights the need to recognize significant challenges related to professional identity in the implementation of CDSS and similar technologies. A better understanding and awareness of individual barriers to CDSS implementation among health professionals can promote the diffusion of such data-driven tools in health care.
- This narrative synthesis maps, interconnects, and reinterprets existing empirical research and provides a foundation for further research to explore the complex interrelationships and influences of perceived professional identity-related mechanisms among health care professionals in the context of CDSS implementations.

## Background

Health care organizations increasingly implement clinical decision support systems (CDSSs) due to rising treatment costs and health care professional staff shortages [1, 2]. CDSSs provide passive and active referential information, computer-based order sets, reminders, alerts, and patient-specific data to health care professionals at the point of care by matching patient characteristics to a computerized knowledge base [1, 3, 4]. These systems complement existing electronic health record (EHR) systems [5] and support various functional areas of medical care, such as preventative health, diagnosis, therapy, and medication [6, 7]. Research has shown that CDSSs can improve patient safety and quality of care [8–10] by preventing medication errors and enhancing decision-making quality [11]. However, despite their potential benefits, their successful implementation into the clinical workflow remains low [1, 12]. To facilitate CDSS acceptance and minimize user resistance, it is crucial to understand the factors affecting implementation success and identify the sources of resistance among the users [1, 13, 14].

In the health care innovation management and implementation science literature, a range of theoretical approaches have been used to examine the implementation and diffusion of health care information technologies. Technology acceptance theories focus on key determinants of individual technology adoption, such as *ease of use*, *perceived usefulness* or *performance expectancy* of the technology itself [15–17]. Organizational theories emphasize the importance of moving beyond an exclusive focus on the acceptance of technology by individuals. Instead, they advocate for examining behaviors and decisions with a focus on organizational structures and processes, cultural and professional norms, and social and political factors such as policies, laws, and regulations [18, 19]. Other studies analyze the implementation of new technologies in health care from a behavioral theory perspective [20] and propose frameworks to explain how and why resistances emerge among users, which may have cognitive, affective, social, or environmental origins [13, 21, 22]. For example, the *Theoretical Domains Framework* has been applied to the behavior of health care professionals and serve as the basis for studies identifying influences on the implementation of new medical technologies, processes, or guidelines [21, 23]. Other, more holistic, implementation frameworks, such as the *Nonadoption, Abandonment, Scale-up, Spread and Sustainability framework*, identify determinants as part of a complex system to facilitate CDSS implementation efforts across health care settings [13].

However, these theoretical approaches do not sufficiently take into account the unique organizational and social system in hospitals, which is characterized by strong hierarchies and the socialization of physicians into isolated structures and processes, making CDSS implementation particularly difficult [5, 24, 25]. Health care professionals are considered to have an entrenched professional identity characterized by the acquisition of a high level of expertise and knowledge over a long period of time, as well as by their decision-making authority and autonomy in clinical interventions. Defined roles and structures of different professional groups in medical organizations help to manage the multitude of tasks under high time pressure [26]. In addition, heath care professionals bear a high degree of responsibility in terms of ensuring medical quality and patient well-being [27]. Changing their professional identity is particularly difficult as they work in organizational contexts with high levels of inertia and long-lived core values based on established practices and routines [27]. This resilience of health care professionals’ identity makes it particularly difficult to implement new technologies into everyday medical practice [28].

By integrating existing evidence into an individual physician’s decision-making processes, CDSSs carry the disruptive potential to undermine existing, highly formalized clinical knowledge and expertise and professional decision-making autonomy [5, 24, 29, 30]. Research has shown that health professionals may perceive new technologies, such as CDSSs, as a threat to their professional identity and draw potential consequences for themselves and their professional community, such as the change of established organizational hierarchies, loss of control, power, status, and prestige [31–33]. Nevertheless, other studies have shown that health professionals view CDSSs as tools that increase their autonomy over clinical decisions and improve their relationship with patients [34, 35]. In addition, these consequences may vary widely by country, professional status, and medical setting. As a result, the use and efficacy of CDSSs differ around the world [24]. We therefore suggest that a better understanding of the identity-undermining or identity-enhancing consequences of CDSSs is needed. Despite growing academic interest, there is surprisingly scant research on the role of perceived identity threats and enhancements across different professional hierarchies during CDSS implementation and how they relate to other human, technological, and organizational influencing factors [5, 36, 37].

Therefore, the purpose of this narrative review is to analyze the state of knowledge on the individual, technological, and organizational circumstances that lead various health professionals to perceive CDSSs as a threat or enhancement of their professional identity. In doing so, this study takes an exploratory approach and determines *human*, *organizational*, and *technological* factors for the successful implementation of CDSSs. Our study extends the current knowledge of CDSS implementation by deconstructing professional identity related mechanisms and identifying the antecedents of these perceived threats and enhancements. It addresses calls for research to explore identity theory and social evaluations in the context of new system implementation [5, 38, 39] by aiming to answer the following research questions: What are the human, technological, and organizational factors that lead different health care professionals to perceive a CDSS as a threat or an enhancement of their professional identity? And, how do perceptions of threat and enhancement of professional identity influence CDSS implementation?

This study is designed to guide medical practice, health IT providers, and health policy in their understanding of the mechanisms that lead to conflicts between health professionals’ identity and CDSS implementation. It is intended to identify practices that may support the implementation and long-term use of CDSSs. By narratively merging insights and underlying concepts from existing literature on innovation management, implementation science, and identity theory with the findings of the empirical studies included in this review, we aim to provide a comprehensive framework that can effectively guide further research on the implementation of CDSSs.

### Understanding professional identity

Following recent literature, professional identity refers to an individual’s self-perception and experiences as a member of a profession and plays a central role in how professionals interpret and act in their work situations [25, 37, 40–42]. It is closely tied to a sense of belonging to a professional group and the identification with the roles and responsibilities associated with that occupation. Professionals typically adhere to a set of ethical principles and values that are integral to their professional identity and guide their behavior and decision-making. They are expected to have specialized knowledge and expertise in their field. In return, they are granted a high degree of self-efficacy, autonomy, and ability to act in carrying out these tasks [25, 43]. In addition, professionals make active use of their identities in order to define and change situations. Self-continuity and self-esteem encourages these professionals to align their standards of identification with the perceptions of others and themselves [44]. Many professions have formal organizations or associations that promote and regulate their shared professional identity [45]. Membership in these associations, adherence to their standards and to a shared culture within their field, including common rituals, practices, and traditions, may reinforce their professional identity [33, 36, 45].

Studies in the field of health care innovation management and implementation science reported a number of professional identity conflicts that shape individual behavioral responses to change and innovation [5, 24, 33, 36, 45, 46]. The first set of conflicts relates to individual factors and expectations, such as their personality traits, cognitive style, demographics, and education. For example, user perception of a new technology can be influenced by professional self-efficacy, which can be described as perceived feeling of competence, control and ability to perform [47]. Studies have shown that innovations with a negative impact on individual’s sense of efficacy tend to be perceived as threatening, resulting in a lower likelihood of successful implementation. Users who do not believe in their ability to use the new system felt uncomfortable and unconfident in the workplace and were more likely to resist the new system [48, 49].

The second set of studies relates professional identity to sense-making, which involves the active process of acquiring knowledge and comprehending change based on existing professional identities as frames of references [50]. For example, Jensen and Aanestad [51] showed that health care professionals endorsed the implementation of an EHR system only if it was perceived to be congruent with their own role and the physician’s practice, rather than focusing on functional improvements that the system could have provided. Bernardi and Exworthy [52] found that health care professionals with hybrid roles, bearing both clinical and managerial responsibilities, use their social position to convince health care professionals to adopt medical technologies only when they address the concerns of health care professionals.

The final set of studies address struggles related to a disruption of structures and processes that lead to the reorganization of the health professions [53, 54] and the introduction of new professional logics [55]. These can result in threat perceptions from the perspective of health professionals regarding their competence, autonomy, and control over clinical decisions and outcomes. Accordingly, the perception of new systems not only influences their use or non-use, but implies a dynamic interaction with the professional identity of the users [56]. CDSSs may be perceived as deskilling or as a skill enhancement by reducing or empowering the responsibilities of users and thereby as compromising or enhancing the professional role, autonomy and status.

Taking the classical theoretical frameworks for the evaluation of health information systems [57] and this understanding of professional identity as a starting point, our narrative review identifies, reinterprets, and interconnects the key factors to CDSS implementation related to threats or enhancement of health professionals’ identity in different health care settings.

## Method

**Fig. 1:**
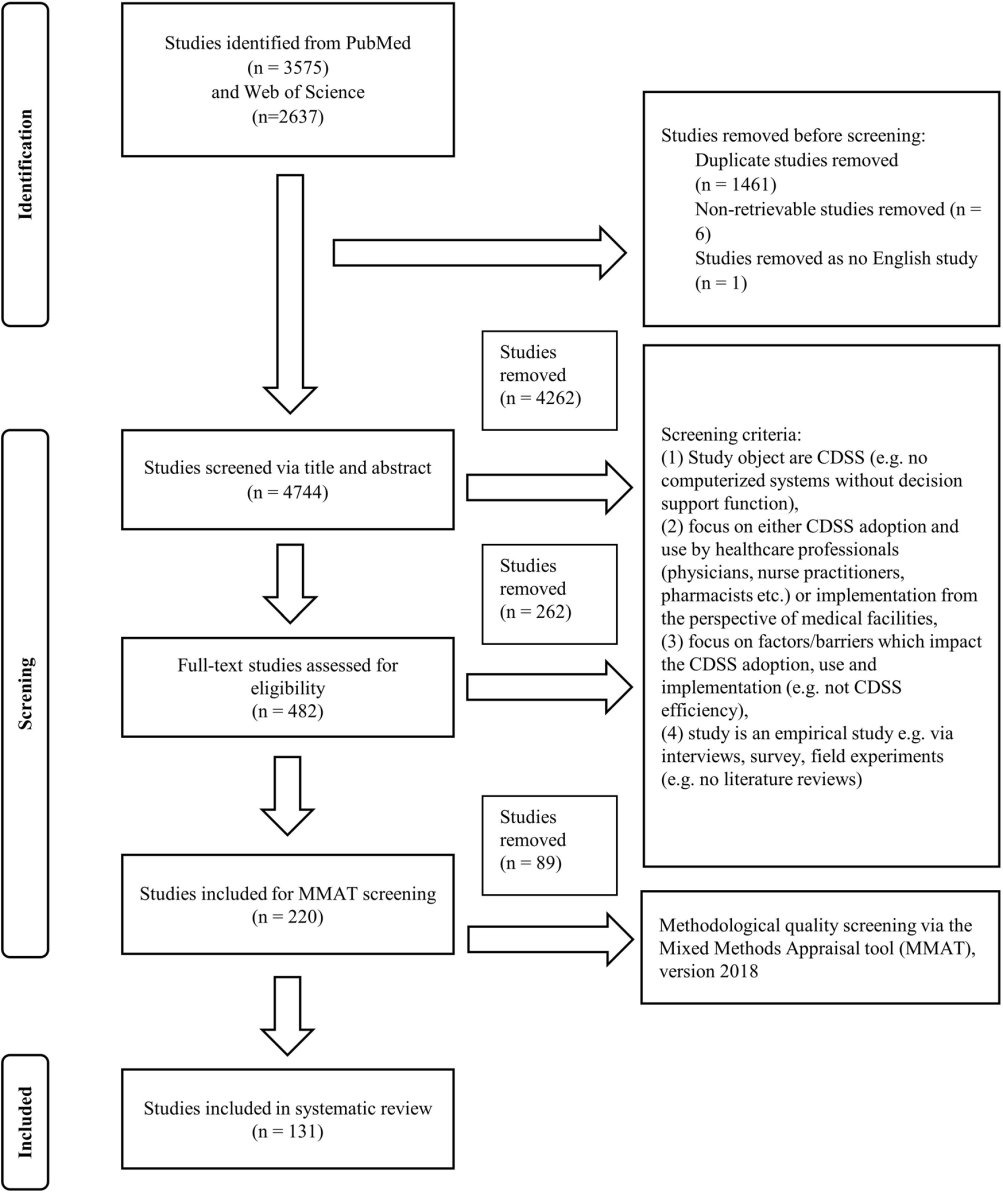
Overview of article screening process

We conducted a comprehensive search of the Web of Science and PubMed databases to identify peer-reviewed studies on CDSS implementations published between January 2010 and September 2023. An initial review of the literature, including previous related literature reviews, yielded the key terms to be used in designing the search strings [1, 49]. We searched for English articles whose titles, abstracts, or keywords contained at least one of the search terms, such as “clinical decision support system,” “computer physician order entry,” “electronic prescribing,” or “expert system.” To ensure that the identified studies relate to CDSS implementation, usage, or adoption from the perspective of health care organizations and health care professionals, we included, for example, the words “hospital,” “clinic,” “medical,” and “health.” The final search strings are provided in Table S1 (Additional file 1). We obtained a total of 6212 articles. From this initial list, we removed 1461 duplicates, 6 non-retrievable studies, and 1 non-English articles. This left us with a total of 4744 articles for the screening of the titles, abstracts, and full texts. Three authors independently reviewed these articles to identify empirical papers which met the following inclusion criteria: (a) evaluated a CDSS as a study object, (b) examined facilitating factors or barriers impacting either CDSS adoption, use or implementation, (c) were examined from the perspective of health care professionals or medical facilities, and (d) represented an empirical study. We identified 220 studies that met our inclusion criteria. The three authors independently assessed the methodological quality of these 220 selected studies using the Mixed Methods Appraisal tool (MMAT), version 2018 [58]. The MMAT can be used for the qualitative evaluation of five different study designs, i.e., qualitative, quantitative, and mixed methods approaches. It is a qualitative scale that evaluates the aim of a study, its adequacy to the research question, the methodology used, the study design, participant recruitment, data collection, data analysis, presentation of findings, and the discussion and conclusion sections of the article [59]. One hundred thirty-one studies were included in the review after excluding studies based on the MMAT criteria, primarily due to a lack of a defined research question or a mismatch between the research question and the data collected [58]. Any disagreement about the inclusion of a publication between was resolved through internal discussion. Figure 1 summarizes our complete screening process.

The studies included in the review were then subject to a qualitative content analysis procedure [60, 61] using MAXQDA, version 2020. For data analysis, we initially followed the principle of “open coding” [62]. We divided the studies equally among the three authors, and through an initial, first-order exploratory analysis, we identified numerous codes, which were labeled with key terms from the studies. Based on a preliminary literature review, we then developed a reference guide with the main categories of classic theoretical frameworks for health information systems implementation (human, technology, organization) [57] and further characteristics of the study. Second-order categories were obtained through axial coding [62], which reduced the number of initial codes but also revealed concepts that could not be mapped to these three categories (i.e., perceived threat to professional autonomy and control). This allowed us to identify concepts related to professional identity. Subsequently, a subset of 10% of the studies was randomly selected and coded by a second coder independently of the first coder [63]. Then, an inter-coder reliability analysis was performed between the samples of coder 1 and coder 2. For this purpose, Cohen’s kappa, a measure of agreement between two independent categorical samples, was calculated. *Cohen’s kappa* showed that there was a high agreement in coding (*k* = 0.8) [64]. We coded for the following aspects: human, organizational, technological, professional identity factor conceptualizations, dependent variables, study type and type of data, time-frame, clinician type sample, description of the CDSS, implementation phase [65], target area of medical care [7], and applied medical specialty. Tables 2, 3, 4, 5, 6 and 7 and Table S2 provide detailed data as per the key coding categories.

### Findings

#### Descriptive analysis

A total of 131 studies were included in our review. In line with recent reviews of CDSS implementation research [6, 14, 57], the reviewed articles are distributed widely across journals (Table 1).

**Table 1:** Journals and their 5-year impact factors

Journal title	No. of articles	5-year journal impact factor (2022)^a^
*BMC Medical Informatics and Decision Making*	25	3.5
*International Journal of Medical Informatics*	15	4.9
*Journal of the American Medical Informatics Association*	8	6.4
*Applied Clinical Informatics*	5	2.9
*International Journal of Environmental Research and Public Health*	3	4.8
*Plos One*	3	3.8
*BMC Family Practice*	3	3.3
*BMJ Open*	3	3.3
*Implementation Science*	2	9.7
*Journal of Medical Systems*	2	5.2
*Applied Ergonomics*	2	3.9
*BMC Health Services Research*	2	3.5
*BMC Primary Care*	2	3.3
*Health Informatics Journal*	2	3.0
*Other journals*	55	

^a^2022 Journal Impact Factor, *Journal Citation Reports*[66]

The examined articles were drawn from 69 journals, 55 of which provide only one article. The *BMC Medical Informatics and Decision Making* and *International Journal of Medical Informatics* published nearly a third of the included studies, with 67 articles overall in medical informatics journals. There are additional clusters in medical specialty-related (33), health services, public health, or health care management-related (12), and implementation science-related (2) journals. The journals’ 5-year impact factor measured in 2022 ranged between 2.9 and 9.7. Of our included articles, 67 were published between 2010 and 2016, while 64 were published between 2017 and 2023.

The review includes a mixture of qualitative (*n* = 61), quantitative (*n* = 40), and mixed methods (*n* = 30) studies. Unless otherwise noted, studies indicated as qualitative studies in Table S2 involved interviews and quantitative studies involved surveys. Interviews with individual health care professionals were the most common data collection method used (*n* = 38), followed by surveys (*n* = 58), and focus group interviews (*n* = 25). Most of the interviews were conducted with physicians (*n* = 60) and nursing professionals (*n* = 23). The studies were performed at various sites and specialties, with primary care settings (*n* = 35), emergency (*n* = 11), and pediatric (*n* = 6) departments being represented most frequently. Forty-five articles researched exclusively physicians and 10 covered nurse practitioners as respondents in their sample. Four studies surveyed pharmacists, one study surveyed medical residents as a single target group, and 20 articles included clinical leaders in addition to clinicians to their sample. Twenty-eight studies were longitudinal, although studying system implementation at one point in time will insufficiently explain the expected impact of the novel system on, e.g., the organizational performance outcomes over time [67]. The studies collected data in 29 different countries, with the most common being the USA (*n* = 41), the UK (*n* = 18), and the Netherlands (*n* = 11).

Included studies were additionally coded according to the implementation phase in which the study was conducted (i.e., *exploration, adoption/preparation, implementation, sustainment* phase) [65]. In 43 of the included studies, the analysis was conducted during the exploration phase, i.e., during a clinical trial or an exploration of the functionality and applicability of a CDSS. Nineteen studies were conducted in the active implementation phase, 15 studies in an implementation adoption or preparation phase, and 46 studies in a sustainment phase (i.e., implementation completed and long-term system use). The revealing studies involved an investigation in multiple implementation phases.

Following Berner’s study [7], we classified the examined CDSSs of the included studies according to specific target areas of care. As such, in 93 articles, CDSSs for *planning or implementing treatment* were studied. Thirty-seven studies examined CDSSs whose goal was prevention or *preventive care* screening. In 31 studies, the functional focus of the CDSSs was to provide specific suggestions for potential *diagnoses* that match a patient’s symptoms. Seventeen CDSSs of the included studies focused on *follow-up management*, 15 studies studied CDSSs for *hospital* and *provider efficiency* care plans and 12 focused on *cost reduction and improved patient convenience* (i.e., through duplicate testing alerts). Most CDSSs supported medication-related decisions and processes, such as prescribing, administration, and monitoring for effectiveness and adverse effects (*n* = 30). An overview of the characteristics of the included studies can be found in Table S2.

In the 131 included studies, we identified 1219 factors, which we categorized into human, technological, organizational, and professional identity threat and enhancement-related factors to implementation (Table 2). The total amount of factors is reported in Table 2 for each of our framework’s dimension and for each of our inferred factor sub-categories. The following section delves into the elements of our framework (Fig. 1), starting with the most commonly identified factors. Finally, the CDSS implementation outcomes are described.

**Table 2:** Summary of CDSS implementation factors

CDSS implementation dimension	CDSS implementation factor	Included study references
Technological (*n* = 532)	System fits into existing clinical workflow and organizational structures (*n* = 67)	[3, 5, 8, 9, 12, 24, 30, 34, 35, 68–125]
	Functionalities meeting users’ needs, incl. display of relevant information, customization (*n* = 58)	[8–10, 24, 35, 68–71, 74, 75, 77, 84, 86, 90–93, 95–99, 101–104, 106, 109–112, 114–116, 119–122, 124, 126–143]
	Design of interface and workflow, intuitive navigation (*n* = 49)	[11, 34, 35, 68, 71, 72, 74, 77, 78, 80, 84, 90, 93, 95, 97, 98, 101–103, 106, 107, 110–114, 117, 121, 122, 126, 127, 131, 132, 134–136, 138, 141, 142, 144–153]
	System’s technical quality and scientific evidence, incl. explainability and transparency of decision outcomes ( n = 43)	[8–12, 68–70, 72, 76, 80, 83, 84, 87, 88, 90, 91, 93, 100, 106, 107, 111, 114, 121, 134, 141, 142, 147–149, 151, 152, 154–160]
	Ease of use ( n = 39)	[9, 10, 17, 24, 30, 34, 68, 69, 71, 73, 74, 76–78, 86, 87, 90, 91, 93, 96, 104, 106, 107, 110, 111, 115, 120, 127, 129, 136, 141, 147, 150, 151, 154, 160–166]
	Irrelevant, inaccurate, excessive alerts (*n* = 36)	[9, 11, 24, 35, 68, 69, 72, 74–76, 90, 91, 96, 98, 101, 103–106, 111, 112, 114, 118, 120, 122, 127, 129–131, 136–138, 144, 164, 167, 168]
	Usefulness of system features and functions, incl. practical guidance and functions meeting complexity of patients’ clinical picture (*n* = 32)	[10, 12, 17, 24, 35, 68, 76, 80, 84, 86, 91, 93–95, 101, 104, 106, 110, 112, 115, 132, 140, 144, 145, 148, 160, 162, 169–173]
	Robust and reliable system (*n* = 30)	[10, 11, 24, 35, 68, 72–74, 84, 86, 88–90, 93, 106, 114, 119, 120, 123, 127, 132, 137, 138, 144, 147, 149, 154, 163, 164, 166]
	System interoperability (*n* = 30)	[10, 30, 35, 68, 71, 75, 78, 93, 94, 96, 100, 102, 104, 112, 114, 115, 119, 124, 126, 127, 131, 134, 137–139, 151–154, 163]
	Technical, IT support (*n* = 30)	[3, 8, 10, 11, 17, 68, 69, 77, 79, 84, 85, 89, 95, 99–101, 104, 116, 117, 139, 143, 144, 146, 147, 153, 163, 167, 170, 171, 174]
	Timely and fast access to relevant information, functions (*n* = 26)	[9–11, 35, 68, 69, 73, 77, 78, 95, 96, 106, 111, 114–116, 122, 131, 135, 137, 140, 144, 147, 150, 163]
	Data privacy and security issues (*n* = 22)	[3, 11, 12, 34, 68, 69, 73, 75, 84, 85, 88, 89, 101, 106, 127, 131, 138–140, 163, 175, 176]
	Data quality, standards, and terminologies (*n* = 22)	[34, 68, 72, 74, 77, 93, 94, 96, 101, 103, 108, 110, 120, 140, 146, 153, 161, 163–166, 177]
	Rigidity of system (functional and interface) (*n* = 17)	[10, 11, 34, 69, 74, 79, 84, 93, 94, 109, 114, 132, 135, 144, 147, 168, 170]
	Value, benefit to end users (*n* = 16)	[9, 79–81, 84, 86, 89, 103, 121, 132, 148, 162, 168, 170, 175, 178]
	Efficiency and time saving potential (*n* = 15)	[10–12, 89, 95, 96, 106, 111, 114, 121, 137, 140, 144, 147, 164]
Organizational (*n* = 287)	Work, time pressure and tension (*n* = 52)	[8, 9, 11, 30, 35, 68, 69, 72, 74–77, 79, 82, 84, 87, 88, 90, 91, 93, 95–99, 101, 102, 104, 106, 110, 111, 114, 115, 118, 121–123, 126, 127, 133, 134, 138, 144, 145, 150–153, 155, 162, 169, 171]
	User training and supervision ( n = 48)	[3, 5, 8, 10, 11, 24, 48, 69, 75–77, 79, 83, 84, 86, 88, 92, 97, 101, 103, 104, 107, 110, 115, 124, 129, 130, 135, 139, 144–146, 150–152, 154, 155, 165–167, 171, 174, 175, 178, 179]
	Internal communication, feedback, collaboration, involvement of end users ( n = 45)	[8, 11, 12, 30, 34, 68–70, 72, 74, 76, 79, 80, 82–84, 87, 88, 92, 101, 103–105, 109–112, 115, 116, 118, 121, 122, 129, 131, 134, 139, 140, 143–145, 150, 163, 167, 169, 174, 178, 180, 181]
	Endorsement, support of management, leadership for change (*n* = 33)	[10, 11, 24, 68–70, 72, 76, 79, 82–85, 92, 94, 100, 104, 115, 116, 126, 129, 143–146, 152, 153, 167, 169, 172, 174, 175, 178]
	Readiness of internal IT infrastructure and hardware (*n* = 32)	[8, 10, 11, 17, 68, 79, 80, 82–86, 88, 95, 109, 110, 134, 139, 141, 144, 146, 147, 151, 154, 155, 170, 171, 175, 179–182]
	Internal (re)organization, change of routines, incl. standardization of processes (*n* = 21)	[24, 70, 75–77, 82, 84, 86, 112, 117, 137, 141, 151, 152, 170, 171, 174, 175, 179, 180]
	Organizational culture, innovation climate and policies, incl. psychological safety (*n* = 20)	[9, 10, 12, 68, 72, 76, 81, 86, 94, 115, 118, 124, 131, 139, 151, 153, 165–168, 174]
	Internal IT competencies and knowledge (*n* = 13)	[72, 74, 83, 84, 91, 93, 121, 123, 126, 143, 155, 166, 174]
	Financial and legal issues (*n* = 13)	[24, 68–70, 77, 112, 117, 118, 128, 151–153]
	Hierarchical boundaries (*n* = 10)	[24, 69, 70, 77, 112, 117, 128, 151–153]
Human (*n* = 197)	Individual attitudes and emotional responses, incl. resistance to change (*n* = 41)	[3, 5, 10–12, 17, 24, 35, 68, 77, 81–84, 86–89, 105, 111, 117, 121, 132, 145, 146, 151–154, 160, 162, 163, 167, 169, 170, 172, 175, 178, 180, 183, 184]
	Experience and familiarity with system (*n* = 35)	[3, 5, 10, 24, 48, 69, 79, 82, 87–89, 98, 104, 105, 108, 111, 114, 127, 131, 133, 135, 138, 139, 144, 147, 154, 158, 168, 170, 178, 182, 183, 185–187]
	Trust in system and underlying rule or algorithm (*n* = 31)	[11, 12, 24, 35, 69, 73, 74, 77, 78, 94, 95, 104, 105, 109, 119, 120, 127, 128, 132, 133, 137, 144, 146, 151, 154, 162, 164, 177, 178, 180, 185]
	Computer illiteracy, IT skills (*n* = 23)	[5, 8, 10–12, 35, 73, 82–84, 89, 90, 100, 120, 143, 145, 152, 160, 177, 181, 183, 188]
	Perceived usefulness (*n* = 22)	[5, 70, 73, 90, 94, 101, 104, 109, 112, 113, 115, 116, 128, 133, 135, 136, 154, 157, 164, 165, 181, 185]
	Perceived potential of patient education and empowerment ( n = 13)	[83, 84, 96, 114, 144, 152, 158, 159, 169, 178, 182, 183]
	Intrinsic motivation, passion, expected effort (*n* = 12)	[30, 73, 75, 93, 101, 104, 109, 110, 115, 134, 140, 151, 161]
	Clinical knowledge and skills (*n* = 10)	[8, 74, 77, 94, 119, 137, 152, 160, 177, 185]
	Perceived potential of training, education of clinicians by system (*n* = 10)	[12, 17, 70, 89, 101, 104, 132, 137, 162, 178]
Benefits (*n* = 93)	System improves effectiveness and efficiency of care (*n* = 34)	[8–11, 17, 68, 69, 72, 73, 77, 80, 81, 89, 97, 100, 107, 109, 115, 116, 121, 134, 135, 141–144, 147, 148, 152, 154, 155, 158, 160, 182]
	System is beneficial to patient safety (*n* = 25)	[8, 10, 11, 17, 24, 69, 74, 80, 81, 89, 91, 94, 96, 102, 104, 106, 121, 126, 137, 144, 152, 154, 159, 175, 178]
	System improves quality of care (*n* = 21)	[8–11, 35, 69, 70, 76, 81, 98, 101, 133, 141, 147, 148, 151, 154, 160–162, 164]
	System prevents prescription and treatment errors (*n* = 13)	[8, 11, 24, 69, 77, 80, 102, 107, 134, 136, 144, 154, 169]
Professional identity threat (*n* = 90)	Perceived threat to professional control, autonomy, responsibilities, role (*n* = 58)	[5, 9, 10, 12, 17, 24, 30, 34, 48, 68, 69, 72, 73, 75, 77, 80–82, 84, 86, 89, 91–94, 97, 98, 102–104, 112–114, 116, 120, 121, 126, 128, 129, 131, 132, 134, 137, 140, 144–146, 154, 155, 162–165, 167, 175, 177, 178, 180]
	Perceived loss of control over patient relationship (*n* = 17)	[12, 35, 73, 81, 89, 93, 101, 104, 111, 114, 115, 129, 136, 137, 140, 162, 170]
	Perceived threat to clinical skill and expertise, incl. risk of overdependence (*n* = 15)	[24, 30, 72, 73, 91, 92, 96, 102, 104, 114, 120, 137, 154, 164, 167]
Professional identity enhancement (*n* = 44)	Perceived enhancement of control of patient relationship, incl. beneficial for patient-provider communication (*n* = 18)	[17, 35, 68, 74, 75, 89, 93–95, 109, 132–134, 140, 141]
	Perceived enhancement of professional control and autonomy, responsibilities, role (*n* = 15)	[24, 34, 35, 72, 91, 92, 104, 115, 137, 155, 161, 169, 178, 180]
	Perceived enhancement of clinical skill and expertise (*n* = 11)	[8, 12, 78, 92, 101, 128, 133, 140, 155, 162, 178]

Factors encountered less than 10 times: Organizational: Champions as mediators between professions and facilitators of knowledge (*n* = 8) [24, 70, 82, 92, 99, 166, 169, 174], Social influence (*n* = 8) [100, 143, 148, 154, 159, 161, 172, 182], System implementation costs (*n* = 4) [89, 94, 165, 174], Organization size (*n* = 4) [104, 118, 150, 189]; Environmental: Governmental policies and guidelines, incl. misleading guidelines and tight control (*n* = 7) [90, 117, 123, 126, 146, 152, 174], National funding (*n* = 3) [10, 123, 174], External competition (*n* = 2) [139, 169], Broadband connection (*n* = 1) [10], CDSS misused for political bargaining (*n* = 1) [123]; Human: Collaboration and trust between all involved (*n* = 7) [5, 10, 24, 86, 97, 134, 174], Age (*n* = 6) [83, 84, 105, 140, 147, 167], Dissatisfaction, experience with legacy system, predecessor CDSS (*n* = 1) [190], Language barriers (*n* = 1) [8], Benefits: System improves communication between clinicians and between clinicians and IT, management (*n* = 9) [10, 24, 34, 35, 89, 122, 140, 144, 147], System is beneficial to cost savings (*n* = 8) [10, 12, 69, 104, 141, 147, 148, 174], Clinicians no longer have to remember everything by heart (*n* = 8) [24, 91, 112, 113, 133, 134, 142, 164]

#### Technological factors

At the technological level, perceptions of threat to professional identity were associated with factors related to the nature of the clinical purpose of the CDSS and system quality, such as *compatibility of the CDSS with current clinical workflows* [68–70], *customization flexibility, intuitive navigation* [71, 72, 126], and *scientific evidence* and *transparency of the decision-outcome* [73, 74, 191]*.* A total of 532 technological factors in 125 included studies were identified. In 21 studies, technological factors were related to study participants’ perceptions of professional identity threat, while in 9 studies these factors were related to perceived professional identity enhancements (Table 3). The exemplary quotes are chosen based on their clarity and representativeness related to the overall themes.

**Table 3:** Examples of review findings relating to technological factors

Author	Professional type	Examples
**CDSS fit into clinical workflow**		
[12]	Physicians	Physicians note that CDSS fit into the clinical workflow is a condition for using CDSSs, otherwise the CDSS is perceived as workflow disruption.
[34]	Nursing professionals	If a CDSS provides recommendation that is discrepant with what user thinks or does not appear to consider patient context, it prompts threat to thinking: “Don’t let a tool overtake critical thinking”.
**Intuitive navigation, customization flexibility, applicability**		
[102]	Physicians	A CDSS has to be intuitive and its information must be short and clear.
[127]	Physicians	Physicians welcome possibility to customize CDSS recommendation and to adjust personal preferences: “I want to be able to set the threshold myself”.
**CDSS’s technical quality and scientific evidence**		
[77]	Junior and senior physicians	Senior physicians demand regularly updated evidence-based CDSS whereas junior physicians prefer quick answers, trust the CDSS and do not necessarily read the source.
[127]	Physicians and nursing professionals	Irrelevant alerts for different user groups and for individual users, with varying needs over time: “It shouldn’t be necessary to override so many alerts; only the sections that apply to us [nurses] should be highlighted”; “… You don’t want to receive that alert over and over again”.

The reviewed studies focused primarily on medication-oriented CDSSs. *Relevance, accuracy, and transparency of the recommendations’ quality and scientific evidence* were found to be crucial for their acceptance and use. “*Irrelevant, inaccurate, excessive, and misleading alerts*” were associated with *alert fatigue* and *lack of trust* [72, 75, 76, 127, 144]. Some senior physicians preferred the provision of *evidence-based guidelines* that would reinforce their knowledge, while others advised junior physicians to override the CDSS recommendations in favor of their own instructions. However, residents tended to follow CDSS recommendations and used them to enhance their confidence about a clinical decision [69, 77, 128]. Physicians had diverse perceptions of the *scientific evidence* supporting the CDSS recommendations. Some regarded it as abstract or useless information that was not applicable to clinical decision making in practice. These physicians preferred a more conventional approach to learning from the “eminences” of their discipline while pragmatically engaging in the “art and craft” of medicine. CDSSs were perceived as increasingly undermining clinical work and expertise among health professionals [24]. In some studies examining AI (artificial intelligence)-based CDSS, *explainability* and *transparency* of the CDSS recommendations played a major role in maintaining control over the therapeutic process [78, 129].

Many studies indicated that the introduction of a CDSS was perceived as a disruptive change to established clinical workflows and practices [12, 79–81, 167]. *The fit of CDSS with standardized clinical workflows* was seen as critical to the CDSS implementation. Senior clinicians preferred their own workflows and protocols for complex patient cases [82]. Geriatricians, for example, considered CDSS recommendations inappropriate for their clinical workflows because geriatric patients are typically multi-morbid and require individualized care [77]. *Intuitiveness* and *interactivity* of the CDSS were found to reduce the perceived threat to professional identity [5], and *customization* and adjustment of alerts based on specialties’ and individual preferences were perceived to increase competence [10, 127, 130]. Physicians considered that successful implementation of the CDSS depends on the integration of existing clinical processes and routine activities and requires collaboration as well as knowledge sharing among experienced professionals [24].

#### Organizational factors

A total of 287 organizational factors in 104 included studies were identified. In 17 studies, organizational factors were related to study participants’ perceptions of professional identity threat, while in 7 studies these factors were related to perceived professional identity enhancements (Table 4). In the included studies, organizational factors influencing professionals’ perceived threat to their identity have been studied from multiple perspectives, such as *internal collaboration and communication* [145, 178], *(top) managers’ leadership and support* [79, 83], *innovation culture* and *psychological safety* [24], *organizational silos* and *hierarchical boundaries* [69, 70], and the *relevance of social norms and endorsement of professional peers* [161].

**Table 4:** Review findings relating to organizational factors

Author	Professional type	Examples
**Collaboration and communication**		
[145]	Physicians and managers	Successful CDSS adoption requires involvement of physicians and nursing professionals in CDSS customization: “… what they [managers] need to do is sit down with the people in each department and work out what are the processes that are critical for that department because the paper processes that have evolved in each individual section of the hospital have been … refined over years … and if you just provide a generic template (there is) no way of replicating any of that.”
[178]	Pharmacist	Communication and collaboration were seen as important for the intervention and for embedding the intervention into routine practice. Pharmacists adopted different ways of communication with clinicians, in order to engage them with the intervention: “It’s difficult, … when’s the best time to approach doctors to discuss things, … when the surgery is not on, they’re on home visits or they’re in meetings, it’s quite a different way of working. So that’s probably one barrier …, so it’d be difficult probably to get everybody together unless you went to the practice meeting on another day.”
**(Top) management and leadership support**		
[72]	Manager	High importance of communication and follow-up on nursing professionals’ roles and tasks; top management needs to reward users for their self-sufficiency and motivation to use CDSS: “… Some people take it and run with it—the medical assistant and doctor are working together, and some just don’t really see it as their job. Part of what physicians have to do is understand they need to make it known to their MA [medical assistant] that this is an expectation. Some doctors have gotten that and some say they can’t or don’t know how to make their MA [medical assistant] do anything.”
[24]	Physicians	CDSSs should not be introduced like an imposition, as it has the potential to affect clinical autonomy and decision-making. If the use of CDSSs is perceived as a top-down order, clinicians will reject it. Strong endorsement from the top management is essential.
[167]	Physicians	Physicians emphasized the role of leadership in overcoming negative perceptions, fear, and resistance to change by highlighting benefits of CDSSs for the patient.
**Innovation culture, climate for innovation, and psychological safety**		
[171]	Junior physicians	A cultural barrier exists where junior health care professionals believe “… that the use of [CDSS] in front of a patient is perceived as being unprofessional. They, therefore, chose not to use devices in plain view. This concern was also raised in relation to senior colleagues considering junior physicians’ use of their mobile device in front of patients or on the ward as being unprofessional.”
[70]	Junior physician	“I think we hit all the 5 rights of CDS on this one. It’s coming at the right time, to the right person, with the right information, using the right channel, and in the right situation…. There is been no interest in turning it off …”
**Organizational silos and hierarchies**		
[69]	Junior and senior physicians	Senior physicians influence the practices of junior physicians. Junior physicians need organizational support in order to adopt CDSSs due to clinical hierarchy.
[77]	Junior and senior physicians	The implementation of CDSSs leads to changes in professional boundaries: “It’s more hierarchical whether or not we look at the recommendations. Often, I look at guidelines, but after my superiors told me to do something else.” (junior physicians) “The nurses put the residents in a somewhat inferior position because of their confidence and their experience….” (senior physician).
[82]	Junior and senior physicians	Senior physicians emphasize that they should not surrender their autonomy to the CDSS whereas junior physicians perceive a sense of greater clinical autonomy when using the CDSS as it reduces their reliance on senior colleagues
[24]	Physicians and nursing professionals	Disputes over power and control between physicians and nursing professionals arise as the CDSS allows widespread access to scientific evidence, which lead to nursing professionals’ control over medical decisions: “… If we want to implement it it’s key to discuss the rules of access for each profession” (surgeon).
**Social norms and endorsement**		
[154]	Physicians and pharmacists	Uncertainty about the accuracy of the CDSS is mitigated by invoking expert: “… if I was recommended by the pharmacists and by ID [infectious disease] and micro [microbiology] then I am more than happy to use [the CDSS]”; “… I want someone from pharmacology or someone that we trust to tell us that the [CDSS recommendations] are accurate if not more accurate than doing it by hand.”
[192]	Physicians	Physicians sought support from colleagues*:* “… probably more important have been colleagues sharing tips and kind of best practice or best use. Those are the most useful...”

The empirical studies showed that the *innovation culture* plays a critical role in driving change in health care organizations. In this regard, resistance to the implementation of CDSSs may be due to a lack of organizational support as well as physicians’ desire to maintain the status quo in health care delivery [24, 70, 75]. Several key factors influenced the implementation in this regard. These included *appropriate timing of the implementation project, user involvement,* and *dissemination of understandable information through appropriate communication channels* [70]. Some studies showed that an innovation culture characterized by interdependence and cooperation promotes social interaction (i.e., a *psychologically safe environment*), which in turn facilitates problem-solving and learning related to CDSS use [193, 194]. For example, nursing practitioners recognized the potential of CDSSs for collaboration in complex cases, which had a positive impact on team and organizational culture development [24].

S*upportive leadership* (e.g., by department leaders) was found to be critical to successful CDSS implementation. This includes providing the necessary resources, such as time and space for training, technical support, and user involvement in the implementation process, which were negatively associated with *perceived loss of control and autonomy* [11, 69, 79, 83, 84, 145, 174]. Involving not only senior physicians but also nursing and paramedical leaders increased the legitimacy of CDSSs throughout the professional hierarchy and helped to overcome the negative effect of low status on psychological safety by flattening hierarchical distances [24, 70, 72]. In contrast, imposing a CDSS on users, led to resistance. Some physicians and nurses felt that the use of the CDSS was not under their voluntary control (i.e., “we have no choice”, “it’s not an option to not use it”) because these systems have become “as essential as … carrying a pen and a stethoscope,” with physicians feeling that they now “are reliant on the CDSS” [10]. In other cases, top-down decisions led to the resolution of initial resistance toward the CDSS [167]. Overall, committed leadership that involved users and transcended professional silos and hierarchies was critical to successful CDSS implementation. In this context, an established hierarchy and culture of physician autonomy impeded communication, collaboration, and learning across professional and disciplinary boundaries [54, 195, 196]. A well-designed CDSS minimized professional boundaries by, for example, empowering nurses and paramedics to make independent treatment decisions [8, 180]. CDSSs thus provided structured means for nonmedical professionals to receive support in their clinical decision-making that was otherwise reserved for professionals with higher authority [34]. Since CDSSs allow widespread access to scientific evidence, they often led to nursing practitioners’ control or oversight of medical decisions, putting junior physicians in an inferior position, and thus providing an occasion to renegotiate professional boundaries and to dispute the distribution of power [24, 77].

In addition, the provision of *sufficient training* and *technical support* were essential to ensure that physicians and nursing practitioners felt confident in using the CDSS and increased their satisfaction with the system [77, 85]. Embedding new CDSSs into routine practice required *communication and collaboration* among professionals with clinical expertise and those with IT expertise [86, 145, 178]. Involving physicians and nursing practitioners in decision-making processes increased their willingness to change their long-standing practice patterns and embrace the newly introduced CDSS [5, 10]. Facilitating the CDSS uptake therefore required legitimization of the system’s designers and exploited data sources [24]. Similarly, the success or failure of CDSSs implementation depended on the ability of the new system to align with existing clinical processes and routine activities. Often, successful adoption was at risk when the implementation was too far away from the reality of clinical practice because those responsible for designing the CDSS poorly understood the rationale for designing the system in a particular way [145].

In addition, some studies indicated that resistance was overcome by communicating the benefits of the CDSS through contextual activities and providing opportunities to experience the system firsthand. Sharing positive implementation experiences and fostering discussions among actual and potential users could bridge the gap between perceptions and actual use [145, 146]. In this regard, *endorsement from* “*respected*” and “*passionate*” *internal change promoters*, such as expert peers, was seen as key to overcoming user resistance [82]. Confirmation from clinical experts that the new system improves efficiency and quality of care was essential for the general system acceptance [154]. Thus, social influence played an important role, especially in the initial phase of system use, while this influence decreased as users gained experience with the CDSS [182].

#### Human factors

A total of 197 human factors in 99 included studies were identified. In 17 studies, human factors were related to study participants’ perceptions of professional identity threat, while in 6 studies these factors were related to perceived professional identity enhancements. Table 5 summarizes the key findings from the included articles, which relate to three factors: *individual attitudes and emotional responses, experience and familiarization with the CDSS*, and *trust in the CDSS and its underlying source.*

**Table 5:** Examples of review findings relating to human factors

Author	Professional type	Examples
**Individual attitudes and emotional responses**		
[81]	Physicians	Physicians express sentiment of apathy toward CDSSs and perception of not being able to “change the tide”.
[154]	Physicians	Physicians express a degree of skepticism toward the use of CDSSs.
[77]	Junior physicians	If CDSS “is not worked on upstream and if it is not ergonomic, it is a disaster and perceived as a real suffering.”
**Experience and familiarization with the CDSS**		
[127]	Physicians & nursing professionals	Perceived barriers related to knowledge regarding CDSSs functions*:* “I had no idea about all these options! Now, I’m a lot more enthusiastic. I’m going to use it right away!”; “I didn’t even know there was a feedback option, never heard of it before.”
[3]	Physicians	Physicians are unfamiliar with sophisticated CDSSs features, “… such as procedures, reminders, and charting templates, and thus do not fully utilize them.”
[82]	Junior and senior physicians	Junior physicians use CDSSs more than senior physicians because they are still learning the clinical area. Senior health care professionals are experienced and familiar with common practices that they do not need CDSSs.
**Trust in the CDSS and underlying rule or algorithm**		
[154]	Physicians	Physicians want to know the functionality of the CDSS’ underlying decision support rule and its limitations, especially in situations of high risk for patient safety: “There’s just a lot of guesswork and I don’t know what happens when someone’s kidneys are suddenly knocked off. I don’t know if it takes that into consideration.”
[128]	Junior and senior health care professionals	Junior health care professionals trust the CDSS recommendations and use them as a “confidence booster” and to “cross-reference” for their decisions, while senior health care professionals rarely use the CDSS because they believe that the CDSS’ and their own knowledge are identical.
[132]	Physicians	Physicians are comfortable following CDSS recommendations if the guideline is perceived as coming from a credible source.
[151]	Nursing professionals	Nursing professionals perceive CDSSs as more trustworthy and precise compared to paper-based assessment.

It is reported in the empirical studies that physicians often failed to fully utilize the features of CDSSs, such as protocols, reminders, and charting templates, because they often *lacked experience and familiarization with the CDSS* [3, 79, 87, 127]. In addition to insufficient training and time constraints, *limited IT skills* were reported as the main reasons [83, 87, 147, 185]. As a result, users interacted with the CDSS in unintended ways, leading to data entry errors and potential security concerns [88]. According to Mozaffar et al. [131], this includes physicians’ tendency to enter incorrect data or select the wrong medication due to misleading data presentations in the system. Inadequate IT skills and lack of user training also contributed to limited understanding of the full functionality of CDSSs. As such, physicians interviewed in one study expressed the lack of knowledge about basic features of a CDSS, including alerts, feedback, and customization options, as a major implementation barrier [127]. Some studies reported that the lack of system customization to meet the personal preferences of users and the lack of system training weakened their confidence in the system and compromised their clinical decision-making autonomy [10, 83, 89, 90, 127, 183].

Some studies indicated that there were *trust issues* among physicians and nursing practitioners regarding the *credibility of the decision-making outcome* [132, 154], the *accuracy of the CDSS recommendations’ algorithm* [146], and the *timeliness of medical guidelines* in the CDSS [127]. Seniors appreciated medication-related alerts but felt that their own decision-making autonomy regarding drug selection and dosing was compromised by the CDSS [74]. However, they tended to use the CDSS as a teaching tool for their junior colleagues, advising them to consult it when in doubt [77, 128]. In some cases, this led to junior physicians accepting CDSS suggestions, such as computer-generated dosages, without independent verification [128, 144, 154].

Several studies indicated that the CDSS introduction elicited different *individual attitudes* and *emotional responses*. More tenured health care professionals were “*frightened*” when confronted with a new CDSS. Others perceived the CDSS as a “*necessary evil*” or “*unwelcome disruption*” [81], leading to skepticism, despair, and anxiety [3, 145, 167]. Younger physicians, on the other hand, tended to be “*thrilled*” and embraced the technology’s benefits [84, 147, 167]. Motivation, enthusiasm, and a “can do” attitude toward learning orientation and skill development positively influenced engagement in CDSS [11, 83, 84, 145, 184].

#### The role of professional identity threat and enhancement perceptions in CDSS implementation

Overall, we found 90 factors in 65 included studies related to perceptions of professional identity threat among the study participants. Forty-four factors in 34 included studies were associated with perceived professional identity enhancements. We identified three key dimensions of professional identity threat and enhancement perceptions among health care professionals impacting CDSS implementation along different implementation phases [197]. Table 6 contains exemplary quotes illustrating the findings.

**Table 6:** Examples of review findings relating to professional identity threat and enhancement

Author	Professional group	Examples
**Threat to professional control and autonomy**		
[154]	Physicians	CDSSs’ potential to substitute physician knowledge is viewed as a threat: “…So, the fact that it [CDSS] can’t take in the whole clinical picture but manually we can.”
[91]	Physicians	“… it [CDSS recommendation] makes me feel useless.”
[128]	Senior and junior physicians	Senior physicians perceive CDSSs as threat to their authority over junior physicians: “Junior physicians were inclined to accept [the CDSS’] recommendations most of the time, but had to override its recommendations when senior colleagues decided on a different antibiotic.”
**Threat to professional skills and expertise**		
[74]	Nursing professionals and physicians	Physicians and nursing professionals become dependent on pharmacists’ knowledge and expertise when resolving complicated CDSS order checks.
[24]	Physicians	CDSSs threaten physicians’ expertise and conscience: “… It’s humiliating to think that we can be substituted by a computer! … We need to have the courage to do what we think is right, not to merely comply with the guidelines dictated by a system. … The knowledge that I get from visiting 150 patients is more substantial than what [the CDSS] can give me.”
[77]	Junior physicians	Even junior physicians acknowledge that if CDSSs are misused or used too much, they “forget to think” and “going to lose the ability to think by ourselves.”
[154]	Physicians	This potential loss of skill was seen as particular problematic in situations in which decision support differs between institutions: “… when we use a lot more programs we don’t think as much, so if we do go to other hospitals where they don’t have these programs then you may not be as well versed in how to dose and adjust vancomycin.”
[24]	Orthopedics	CDSSs are perceived as not being a useful tool for orthopedic specialties: “The actual evidences in [orthopedic surgery] are not very many, you know, I can’t really see how [the CDSS] would be useful for us. …. The actual tools of an orthopedic resemble those of a crafts worker. … We learn by reading books and articles, but also by … observing the experts at work, learning how they do things...”
**Loss of control over patient relationships**		
[162]	Physicians	Physicians stated that they “… are responsible for the treatment of their patients and not a CDSS.”
[81]	Physicians	Physicians stated that “the problem with all of this (digitization) is that it is so impersonal. It takes all the joy out of practicing medicine. I want to build a relationship with the patient. It isn’t all about the medication, they want to share their pain, anxiety, family issues. We can’t change the tide. We can’t do anything about this (the move to digital).”
**Enhancement of professional control and autonomy**		
[34]	Nurse practitioners	Nurse practitioners perceived the CDSS as an empowerment: “… If a CDS tool is designed well, it could empower nurses to advocate for patients and contribute to treatment decision-making. As an objective assessment of a patient’s condition, the CDS tool has the potential to provide participants with a structured method by which nurses can garner support for their recommendations.”
[8]	Nurse practitioners	The CDSS “empowered staff nurses to manage more complicated scenarios independently.”
[155]	Physicians	The care professionals expressed that the CDSS could enhance their control and confidence in their work: “Off hand, I would say that I would get a better feeling of what I do – and an overview of the patients, especially when we take over each other’s patients.”
**Enhancement of professional skills and expertise**		
[162]	Physicians, nurse practitioners	“… sixty-two percent of the respondents reported that advice of a CDSS on how to treat a (…) patient is a welcome supplement to their own expertise, …”
[178]	Pharmacist	Pharmacists saw the CDSS as: “offering opportunities to demonstrate their skills and to further develop their role working within general practice settings.”
[24]	Physicians	Physicians viewed the CDSS as a useful tool, but not to support their own work, but as a support tool for other specialists or residents with less clinical experience: “Maybe I could use it. I think it would be more useful for young physicians, those who have only just graduated, or those with little experience… You know, to avoid mistakes…” “It’s brilliant. Really, really useful. I think it’s more so for medics though, rather than [surgeons].”
**Enhancement of control over patient relationships**		
[35]	Physicians	Physicians expressed the need for CDSS features which enhance patient communication, such as “informative yet brief patient summaries” as this would provide them with a “greater sense of control” over the digitalized information and knowledge exchange with patients, and engender greater trust between patients and physicians.

A number of physicians perceived CDSSs as an ultimate *threat to professional control and autonomy*, leading to a potential deterioration of professional clinical judgment [30, 69, 77, 154, 155]. Most nurse practitioners, on the other hand, experienced a shift in decision-making power, providing an occasion to renegotiate professional boundaries in favor of health care professionals with lower levels of expertise [24]. Thus, nurses associated the implementation of a CDSS with *enhanced professional control and autonomy* in the performance of tasks [34, 155, 169]. Pharmacists often advocated for medication-related CDSSs, which in turn increased physician dependency and resistance to new tasks [12, 84, 178]. The latter was a consequence of physicians’ increasing reliance on pharmacists for complex drug therapies, as physicians had to relinquish some decision-making authority to pharmacists by restructuring of decision-making processes [74].

Senior physicians frequently expressed concerns about *overreliance on CDSS* and *potential erosion of expertise*, which they believed led to *patient safety risks* [10, 24, 75, 89, 155]. They complained that overreliance on CDSS recommendations interfered with their cognition processes. For example, in medication-related CDSSs, clinical data such as treatment duration, units of measure, or usual doses are often based on pharmacy defaults that may not be appropriate for certain patients. According to these physicians, their junior colleagues might not double-check recommended medication doses and treatment activities, leading to increased patient safety risk [131]. In another study, general practitioners expressed concerns about the deskilling of future physicians through CDSSs. Some CDSSs required a high level of clinical expertise, skill, and knowledge regarding the correct entry of clinical information (e.g., symptoms) for proper support in clinical decisions. Many physicians feared that the use of CDSSs would erode this knowledge and thus allow the CDSS recommendations to lead to incorrect decisions [30]. This potential *loss of skills and expertise* was seen as particularly problematic in situations where decision support for medications and e-prescriptions varied from facility to facility. Physicians working at different institutions who relied on the CDSS for medication treatment support used at one institution reported that they had difficulties making the correct clinical decisions at the other institution [154]. From the reviewed articles, it appeared that senior physicians perceived CDSSs as an intrusion into their professional role and object to their expertise and time being misused for “*data entry work*” [10]. They enjoyed the freedom to decide what to prescribe, when to prescribe it, and whether or not to receive more information about it [77] and were determined not to “*surrender*” and “*be made to use [the CDSS]*” [82].

In line with the increasing dependence of physicians on pharmacists when using CDSS for medication treatment, pharmacists used the CDSS to demonstrate their professional skills and to further develop their professional role [178]. Nurse practitioners were empowered by CDSSs guidance to systematically update medications and measurements during their hectic daily clinic routine [24, 91], to independently manage more complicated scenarios [8], and to facilitate their decision-making [92]. Some physicians stated that CDSS recommendations facilitated their critical thinking to critically reflect on the medication more than usual and facilitated more conscious decisions [133]. Increased *professional identity enhancement in terms of skills and expertise* were thus often associated with technological factors such as enhanced patient safety, improved efficiency, and quality of care [9].

Furthermore, physicians strongly associated their professional identity with their central role in the quality of patient care based on a high level of empathy and trust between physician and patient [45, 195]. Their perceived threat to professional identity lead to a *sense of loss* in clinical professionalism and *control over patient relationships* [162, 170]*.* CDSS usage was perceived as unprofessional or disrupting to the power dynamic between them and their patients [89, 93, 171]. As a result, they indicated that established personal patient relationships were affected by imposed CDSS use [81]. Other physicians saw CDSSs as having potential to *enhance patient relationships* providing them with more control over the system and treatment time, facilitating information and knowledge sharing with patients and building trust between patients and physicians [35, 94].

Mapping the perceptions of threat and enhancement of professional identity among physicians and other health care professionals identified in each study to implementation phases allowed for an examination of the evolution of identity perceptions in CDSS implementations. Table 7 assigns the identity perceptions among physicians and other health care professionals to the different implementation phases. The findings illustrate that threat perceptions were predominantly perceived before and at the beginning of implementation. With steady training, use and familiarization with the CDSS, the perceived threat to professional identity slightly decreased in the sustainment phase, compared to the pre-implementation phase, while perceptions of enhancement of professional identity increased. During the exploration phase, physicians in particular perceived the CDSS as undermining their professional identity, and this perception remained relatively constant through the sustainment phase. Other health care professionals, such as nurse practitioners and pharmacists often changed their perspective over the course of the implementation phases and perceived the CDSS as supporting their control, autonomy, and skill enhancement at work.

**Table 7:** Table illustrating professional identity threats and professional identity enhancements as perceived by health care professionals across implementation phases

Author	Professional group	Examples
**Perceived professional identity threats**		
**Exploration phase**		
[1]	Physicians	“… the more reliant we become on technology even with [the CDSSS] and things you de-skill a bit.”; “… the clinical judgement aspect of prescribing vancomycin will go down.”
[2]	Physicians	“You want to be free to decide what you are prescribing, when you are prescribing it and you want to be free to decide if you are going to get the information or not.”
[3]	Physicians	“The digital clinic that steal our patients, we experience that.”
[4]	Physicians	“[Physicians] were concerned about the deskilling of future doctors through the use of [CDSSs].”
[5]	Physicians	“Clinical decision making is still my primary role, like, so it’s up to me.“
[6]	Physicians and nurse practitioners	“I mean, I know it’s not mandatory to follow the recommendations, but it still feels that way. Sometimes, you’re just happy that somebody is using the medication you prescribe at all, and then you get the recommendation to switch the medication. [The CDSS] seems to always tell you that it’s not good enough. It’s never good enough.”
[7]	Physicians	“I am opposed to [the CDSS], as I see it as another task being delegated to physicians that can better be done by those trained and experienced in it. I would prefer to concentrate on those things I do well rather than spending time doing secretarial work. Some of us do not round frequently in the hospital anymore, which will make staying competent in the system difficult…”
[8]	Physicians	“The computer system should be allowed to block you. I have my reasons to do what I do and maybe I will think about its suggestions, but I do not want [the] IT [department] to block me at those moments. … I always want to do what I want.”
[9]	Physicians	“CDSS technology enforces strict working according to guidelines and thus may deprive physicians from their sense of added value. This (…) makes physicians feel less valuated.”
[10]	Nurse practitioners	The nurse practitioners complained that “…critical thinking [is lost] once the tool is embedded into [the] workflow.”
**Adoption decision, implementation preparation, active implementation phase**		
[11]	Physicians and other healthcare professionals	Physicians and nurse practitioners mentioned being threatened in their own clinical practice and autonomy and they were reluctant to use a CDSS when it interfered too much with clinical practice: “When the CDSS becomes leading and the clinical view of the practitioner is subordinated”, “When my role as a care provider is undermined or becomes more complicated.”, and “I would like to keep my own clinical reasoning without a CDSS.”
[12]	Physicians, pharmacists, general practice staff	“Pharmacists saw the dashboard component as offering opportunities to demonstrate their skills and to further develop their role working within general practice settings.”; “I think it’ll give us a useful tool to be able to perhaps design our programmes of work, and also thinking about if we’re going to run any quality programmes in the future, it will hopefully help us to design what we’re working on because it will give us that information, give us that baseline that we need so often.”
**Sustainment phase**		
[13]	Cardiologists, heart failure nurses	“Seventy-nine percent stated that they are responsible for the treatment of ‘their’ patients and not a CDSS.”
[14]	Physicians	“The professionals who participated in this study’s in-depth interview were dissatisfied with this integrated management system and wanted the ability to customize and adjust the alerts they received.”
[15]	Nurse practitioners	“… I should be able to order that if I think it’s indicated without needing further approval.”
**Perceived professional identity enhancements**		
**Exploration phase**		
[16]	Physicians and nurse practitioners	The physicians expressed that the CDSS could enhance their control and confidence in their work: “Off hand, I would say that I would get a better feeling of what I do – and an overview of the patients, especially when we take over each other’s patients.” (physician); Nurses appreciated the CDSS recommendations, protocols and checklists to support monitoring activities: “I think it would be great to know what is recommended because we have tuberculosis patients” (nurse practitioner).
[10]	Nurse practitioners	“If a [CDSS] is designed well, it could empower nurses to advocate for patients and contribute to treatment decision-making.”
[6]	Physicians and nurse practitioners	“We think that the traditional treatment relationship between patient and clinician is fundamentally changing, it is becoming more horizontal, not in every aspect but in many. That is where it is supposed to go. I really think [the CDSS] can facilitate this because it increases commitment and a feeling of ownership.”
**Adoption decision, implementation preparation, active implementation phase**		
[20]	Physicians and nurse practitioners	“As a consequence of a reminder for drug dosing in renal malfunction, I reduced the methotrexate dose, which I had forgotten” (physician); “Once when my doctor was away, I used the warfarin assistant to define the dosing” (nurse practitioner).
[21]	Physicians	“[The CDSS] is integrated in the workflow because after talking with the patient, the physician always returns to the computers and goes into the EHR. The [CDSS] fits in this workflow. If the physician is unsure on what to order, they will go to [the CDSS].”
[22]	Pharmacists	“Despite the fact that these evaluations would represent an added responsibility, pharmacists felt that this was in line with why they chose the profession in the first place, and welcomed any [CDSS] that would increase their role in patient care.”
**Sustainment phase**		
[13]	Cardiologists, heart failure nurses	“A total of […] 55% stated that a CDSS supplements their independency as a [heart failure] care expert.”
[23]	Nurse practitioners	“Some nurses thought that [the CDSS] could supplement their clinical reasoning to facilitate decision-making; …”
[24]	Nurse practitioners	“After the implementation of the CDSS, we are now more focused on the kind of food we order for the residents”, and “When screening a new resident, I can see from using the CDSS the new interventions that are necessary, what we can work on and what can wait.”

#### CDSS implementation outcomes

In total, we identified 93 benefits related to CDSS implementation in the reviewed studies (Table 2). The most commonly evaluated benefits were *improvements in work efficiency and effectiveness* through the use of CDSSs, *improvements in patient safety, and improvements in the quality of care*. *Prevention of prescription and treatment errors* was also frequently mentioned. The included studies measured CDSS implementation in various ways, which we classified into seven groups (Table 8). Most studies measured or evaluated *self-reported interest in using the system or intention, willingness to use, or adoption*, followed by *self-reported attitude toward CDSSs*, and *both self-reported and objective measure of implementation success*. *Objective actual use measurement* was evaluated in only 10 studies, while *self-reported use* was measured in seven studies, and *self-reported satisfaction and performance of the system* was measured in five studies. *Both self-reported* and *objective measure of usefulness and usability* was measured in one study.

**Table 8:** Approaches used to measure CDSS implementation in the 131 studies

Measurement approach and number of studies	Included study references
Self-reported interest in using or intention, willingness to use, adoption (*n* = 74)	[5, 8, 9, 12, 17, 48, 69, 72, 73, 77, 79, 82–84, 87, 90, 91, 93, 95, 96, 100, 105, 106, 108, 109, 111, 113, 116–118, 121, 124, 127, 129–135, 138–141, 143, 149, 151, 153–155, 157–159, 162, 163, 165, 166, 168–172, 174, 177–180, 182, 183, 187, 189, 198–200]
Self-reported attitude toward using CDSS (*n* = 29)	[24, 30, 35, 72, 75, 80, 85, 92, 98, 100, 102, 103, 108, 115, 120, 122, 126, 128, 136, 147, 148, 160, 161, 164, 167, 175, 181, 184, 185]
Self-reported use (*n* = 7)	[11, 34, 68, 92, 112, 158, 190]
Self-reported satisfaction, performance (*n* = 5)	[107, 112, 122, 142, 147]
Both self-reported and objective measure of implementation success (*n* = 21)	[35, 70, 76, 78, 86, 88, 91, 94, 97, 99, 104, 110, 114, 131, 139, 144, 146, 156, 174, 178, 180]
Both self-reported and objective measure of usefulness, usability (*n* = 1)	[119]
Objective actual use measurement (*n* = 10)	[74, 89, 92, 96, 102, 104, 137, 186, 188, 189]

Although we included 40 quantitative studies in our review, only a few of these empirically measured the direct effect of professional identity threat or related organizational consequences on implementation, adoption, or use of CDSSs. Two studies empirically demonstrated a direct significant negative relationship between perceived professional autonomy and intention to CDSS use [5, 48]. Another four studies found empirical evidence of an indirect negative association between threats to professional identity and actual CDSS use. Physicians disagreed with the CDSS recommendation because they perceived insufficient control and autonomy over clinical decision making [79, 88] and lacked confidence in the quality of the CDSS and its scientific evidence [154].

## Discussion

### Main findings

**Fig. 2:**
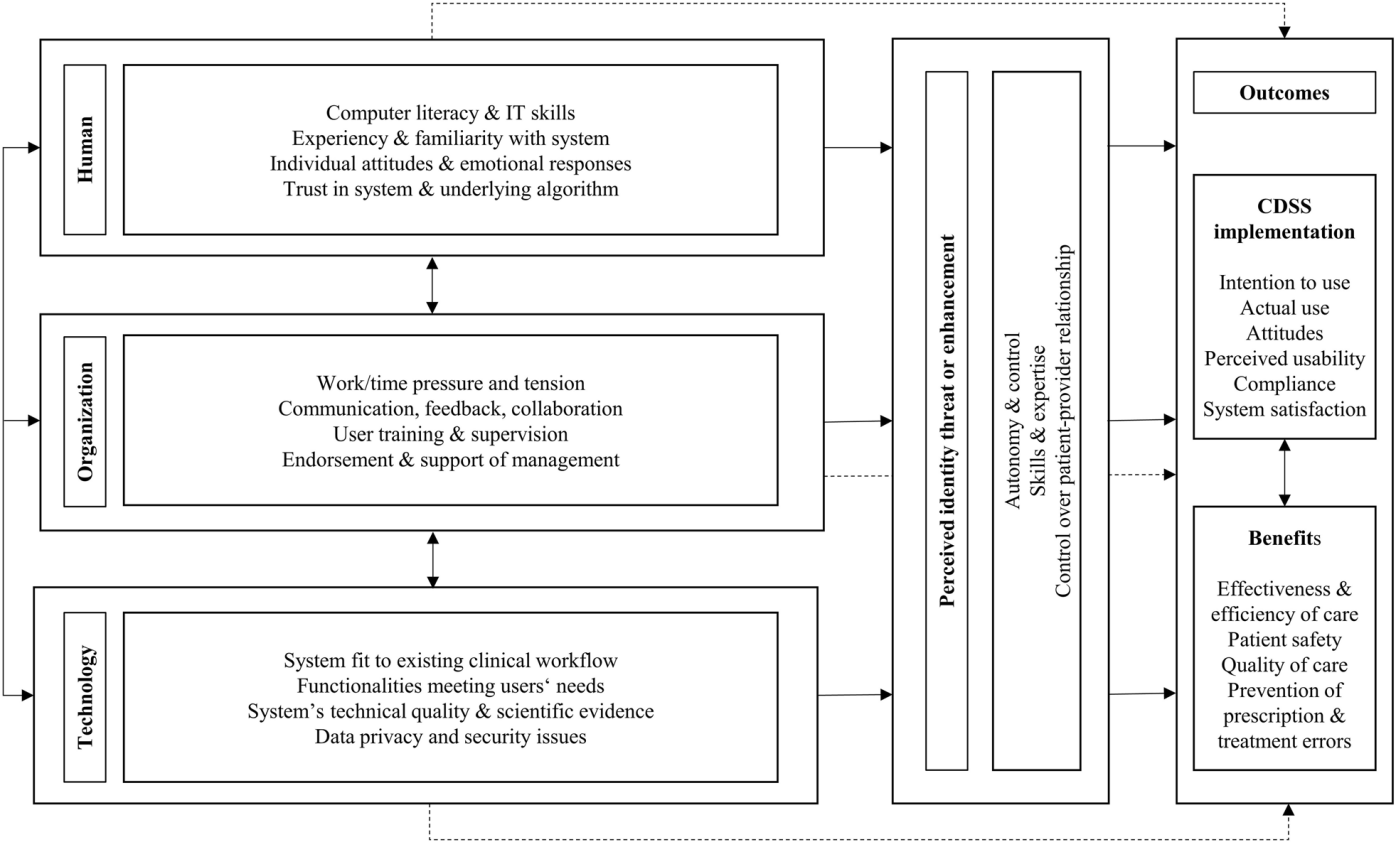
A framework for the role of professional identity in CDSS implementation

The purpose of this narrative review was to identify, reinterpret, and interconnect existing empirical evidence to highlight individual, technological, and organizational factors that contribute to professional identity threat and enhancement perceptions among clinicians and its implications for CDSS implementation in health care organizations. Using evidence from 131 reviewed empirical studies, we develop a framework for the engagement of health care professionals by deconstructing the antecedents of professional identity threats and enhancements (Fig. 2). Our proposed framework highlights the role of cognitive perceptions and response mechanisms due to professional identity struggles or reinforcements of different individual health care professionals in the implementation of CDSSs. Our work therefore contributes to the growing literature on perceived identity deteriorations with insights into how knowledge-intensive organizations may cope with these threats [37, 45, 46]. We categorized clinicians’ professional identity perceptions into three dimensions: (1) *perceived threat and enhancement of professional control and autonomy*, (2) *perceived threat and enhancement of professional skills and expertise*, and (3) *perceived loss and gain of control over patient relationships*. These dimensions influenced CDSS implementation depending on the end user’s change of status and expertise over the course of different implementation phases. While senior physicians tended to perceive CDSSs as undermining their professional identity across all implementation stages, nurse practitioners, pharmacists, and junior physicians increasingly perceived CDSS as enhancing their control, autonomy, and clinical expertise. Physicians, on the other hand, were positive about the support provided by the CDSS in terms of better control of the physician–patient relationship. In most studies, professional identity incongruence was associated with technological factors, particularly the lack of adaption of the system to existing clinical workflows and organizational structures (i.e., process routines), and the fact that CDSS functionalities have to meet the needs of users. The lack or presence of system usability and intuitive workflow design were also frequently associated as antecedents of professional identity loss. The other dimensions (i.e., human and organizational factors) were encountered less often in relation to professional identity mechanisms among health care professionals. Only six studies found empirical evidence of an indirect or direct negative relationship between health professionals’ perceived threats to professional identity and outcomes of CDSS implementation, whereas no study explicitly analyzed the relationship between dimensions of professional identity enhancement and outcomes of CDSS adoption and implementation.

### Interpretations, implications and applicability to implementation strategies

The results indicate that healthcare professionals may perceive CDSSs as valuable tools for their daily clinical decision-making, which can improve their competence, autonomy, and control over the relationship with the patient and their course of treatment. These benefits are realized when the system is optimally integrated into the clinical workflow, meets users’ needs, and delivers high quality results. Involving users in design processes, usability testing, and pre-implementation training and monitoring can increase user confidence and trust in the system early in implementation and lead to greater adoption of the CDSS [146]. To address trust issues in the underlying algorithm of the CDSS, direct and open communication, transparency in decision-making values, and clinical evidence validation of the CDSS are crucial [154]. CDSS reminders and alerts should be designed to be unobtrusive to minimize the perceived loss of autonomy over clinical decisions [77].

Contrary, the implementation of a CDSS often lead to substantial changes of professional identity and thereby often associated with fear and anxiety. A sense of a loss of autonomy and control was linked to lower adoption rates and thus implementation failure. Cognitive styles, which may be expressed in emotional reactions of users toward the CDSS, reinforced reluctance to implement and use the system [145, 167]. This underscores the importance of finding expert peers and professionals who are motivated and positive toward CDSS adoption and use, and who can communicate and promote the professional appropriateness and benefits of the CDSS to their colleagues [82, 83, 184]. This promotes a focus on the improvement and benefits of the CDSS while maintaining the integrity, perceived autonomy, control, and expertise of physicians and nurses.

Accordingly, the included studies show that health professionals respond to the professional identity threat triggered by the CDSS implementation by actively maintaining, claiming, or completely changing their identity [39], which is consistent with previous studies elaborating on the self-verification of professionals [44]. For example, physicians delegated routine tasks to other actors to maintain control over the delivery of services and thereby enhance their professional status [201]. Pharmacists used the introduction of CDSS for drug treatment to demonstrate their skills to physicians and to further develop their professional role [178]. Maintaining authority over the clinical workflow without the need for additional relational work with lower-status professionals was seen as one of the main factors for health care professionals’ CDSS acceptance in our findings [10, 12, 84, 178]. Physicians influence change processes, such as the implementation of CDSS, in a way that preserves the status quo of physicians’ responsibilities and practices. They often stated their objective to avoid increasing dependence on lower-status professionals such as nurses or pharmacists who were gaining control by using the new CDSS. In addition, CDSS users frequently criticized the system’s lack of fit with clinical work processes and that the systems were not able to replace the clinical expertise and knowledge [12, 34, 77, 82]. The loss of control over the patient-physician relationship also represented a key component of identity undermining through the introduction of CDSSs. Many physicians expressed that their trust-building interaction with patients was eroded by the functionalities of the CDSS [81, 170]. The fact that the use of CDSSs saves time in patient therapy and treatment, freeing up time for their patients, was rarely expressed [12, 147]. This underscores the need to cope with the physician’s strong identification with their professional role, their tendency to preserve the status quo, and self-defense against technological change during the implementation of CDSSs.

Furthermore, the reviewed studies emphasized the importance of both inter- and intra-professional involvement, collaboration, and communication in health care organizations, during the CDSS implementation, suggesting that these mechanisms influence the extent and quality of cooperative behavior, psychologically safe environments, and role adaptation of different professional groups [26, 54, 55, 202]. Among the studies we reviewed, managerial support and collaboration influenced coordination during CDSS implementation [82, 83, 174], such as by providing usability testing and time for efforts to change the understanding of why and how health care professionals should modify their routine practices [74, 95].

Overall, the review shows that the consideration of perceived professional identity mechanisms among health care professionals plays an important role when implementing new CDSSs in health care organizations. Additionally, perceived threats and enhancements of professional identity should be considered and regularly assessed in long-term oriented implementation strategies. These strategies often include methods or techniques to improve the adoption, implementation, and sustainability of a clinical program or practices [203] and may span from planning (i.e., conducting a local needs assessment, developing a formal implementation plan) to educating (i.e., conduct educational meetings, distribute educational materials) to restructuring professional roles to managing quality (i.e., provide clinical supervision, audit, and feedback) [204, 205]. To ensure implementation, health care professionals of all hierarchies should be involved in the planning and decision-making processes related to CDSS implementation. Continuous feedback loops between health care professionals, IT staff, and implementation managers can help identify unforeseen threats to professional identity and necessary adjustments to the implementation plan. The review found that perceived identity threats particularly need to be addressed among highly specialized physicians to account for their knowledge-intensive skills, expertise, and clinical workflows [24, 96]. In addition, the purpose of CDSS implementation and information about how it aligns with organizational strategic goals and individual professional development should be clearly and continuously communicated at all stages of implementation.

Our review also confirms that health care professionals’ perceptions of the effectiveness of CDSSs reinforce the impact of organizational readiness for the ongoing and required transformation of healthcare [17]. Comprehensive assessments of the suitability of the system for established or changing clinical workflows and the technical quality of the CDSS should be prioritized at the beginning of the implementation. Training programs should be developed to help professionals adapt to the new medical systems and allay fears of a loss of competence or relevance. To mitigate threats to professional identity in the long term, it is necessary to foster an organizational culture of adaptability, learning, and psychological safety, in which it is acceptable to make mistakes and learn from them. In addition, ongoing leadership support and professional development opportunities are critical to ensure that health care professionals continue to adapt their roles and keep pace with technological developments [79, 84].

### Limitations

A literature review of a large sample of empirical studies has many advantages [206]. However, some limitations arise from the study design. First, our included studies were mainly conducted in the USA or UK (see Table S2). The dominance of these two countries may pose a potential bias, as different cultures may have different implications for CDSS implementation and threat perceptions among health care professionals. Therefore, there is a need for caution in generalizing the findings on the impact of human, technological, and organizational factors on professional identity perceptions among professionals across different cultures. More studies are needed to provide a nuanced understanding of professional identity mechanisms among health care professionals across a broader range of cultures and countries.

Second, broad search terms were used to identify a larger number of articles in the literature review and to identify professional identity based on implementation and adoption factors mentioned in the included studies from the perspective of health professionals who were not specifically identified as threats to or enhancements of professional identity. This could also be considered a methodological strength, as this review combines findings from qualitative, quantitative, and mixed methods studies on this construct from a large and diverse field of research on CDSS implementation. However, non-English language articles or articles that did not pass the MMAT assessment may have been overlooked, which would have provided valuable information on further barriers and facilitators (i.e., threats to professional identity in different cultures), affecting the rigor of this study.

Third, most of the studies reviewed captured CDSSs for use in primary care settings. CDSSs in highly specialized specialties or those that frequently treat multi-morbid patients, such as cardiology and geriatrics, require features that allow for detailed workflow customization. In such specialties, even more attention needs to be paid to balancing provider autonomy and workflow standardization [97]. As such, future research should provide the missing evidence in such complex settings.

Fourth, we were only able to identify a limited number of studies that empirically analyzed the causal relationships included in our framework. There is a lack of studies that use longitudinal research designs, quantitative data, or experimental study designs. Therefore, the identified effects of technological, organizational, and human factors on professional identity and consequently on implementation success need to be interpreted with caution. Future research should test whether the determinants and effects of professional identity mechanisms among healthcare professionals can be observed in real-world settings.

## Conclusion

Professional identity threat is a key cognitive state that impedes CDSS implementation among various health care professionals and along all implementation phases [31, 45]. Health care managers need to engage in supportive leadership behaviors, communicate the benefits of CDSSs, and leverage supportive organizational practices to mitigate the perception and effect of professional identity threat. An innovation culture needs to support the use of CDSSs and top management commitment should reduce uncertainty about why a new CDSS is needed [24]. Therefore, leaders should raise awareness of the relevant CDSS functionalities and communicate the terms and conditions of use. It is crucial to involve clinicians in updating CDSS features and developing new ones to ensure that CDSSs can be quickly updated to reflect rapid developments in guideline development [195]. One way to achieve this is to engage proactive, respected, and passionate individuals who can train colleagues to use the CDSS and promote the potential benefits of the system [70, 82].

Our framework presented in this study provides a relevant foundation for further research on the complex relationship between human, technological, and organizational implementation factors and professional identity among different health care professionals. The findings also guide health care management experts and IT system developers in designing new CDSSs and implementation strategies by considering the ingrained norms and cognitions of health care professionals. As suggested above, more research is needed to determine whether some barriers or facilitators are universal across all types of CDSSs or whether there are domain-dependent patterns. In this context, research that explicitly focuses on AI-based CDSSs becomes increasingly important as they become more relevant in medical practice. In fact, five of the studies included in our research, conducted over the last 3 years, examined factors related to the adoption and implementation of AI-based CDSS [73, 74, 96, 205, 206]. AI-based CDSSs extend to full automation and can discover new relationships and make predictions based on learned patterns [97]. However, with their opaque and automated decision-making processes, AI-based systems may increasingly challenge professional identity as they increasingly disrupt traditional practices and hierarchies within healthcare organizations, posing a threat to professional expertise and autonomy [156]. This may further hinder the implementation and sustainable use of these systems compared to non-AI-based systems. Future research could examine overlaps in barriers and facilitators between CDSSs and AI-based systems, which are of relevance for professional identity threat perceptions among health care professionals, and assess the reasons behind these differences. In addition, translating the findings for different medical contexts may provide valuable insights. This can eventually lead to guidelines for the development of CDSS for different specialties.

Some factors were found less frequently during our analysis; in particular, communication of the benefits of a CDSS to users, the importance of trust across different hierarchies and among staff involved in implementation, and government-level factors related to the environment. While the former factors represent important psychological safety and acceptance of the CDSS, the level of the environment represents a minor role in the perception of professional identity. Future research is needed, however, to determine whether all of these factors play an important role in CDSS implementation. Furthermore, future research could explore the role of middle managers and team managers in health care organizations rather than the role of senior management in managing professional identity threats when leading change. Our narrative review found that clinical middle managers may have a special role in legitimizing CDSSs [156]. In addition, a future research opportunity arises from the perceived role and identity enhancement through new technologies and their consequences for social evaluation in hierarchical healthcare organizations [35, 132, 155].

Overall, the findings of this review are particularly relevant for managers of CDSS implementation projects. Thoughtful management of professional identity threat factors identified in this review can help overcome barriers and facilitate the implementation of CDSSs. By addressing practical implications and research gaps, future studies can contribute to a deeper understanding of the threat to professional identity and provide evidence for effective implementation strategies of CDSSs and thus for a higher quality and efficiency in the increasingly overburdened health care system.

### Supplementary Information

**Additional file 1: Table S1. **Final search strings used to identify articles for the review. **Table S2.** Characteristics of included studies.

## Data Availability

The datasets used and/or analyzed during the current study available from the corresponding author on reasonable request.

## Abbreviations

AI: Artificial intelligence
CDSS: Clinical decision support system
EHR: Electronic health record
MMAT: Mixed Methods Appraisal tool
