# Supplementary material for: Exploring the role of professional identity in the implementation of clinical decision support systems—a narrative review
Source: Implement Sci. 2024 Feb 12;19:11. doi: 10.1186/s13012-024-01339-x (PMC10860285; doi:10.1186/s13012-024-01339-x)
Supplement: Supplementary file 1 — Additional file 1: Table S1. Final search strings used to identify articles for the review. Table S2. Characteristics of included studies. [file 13012_2024_1339_MOESM1_ESM.docx]

# Additional file 1.

[Table S1](#Table_A1). Final search strings used to identify articles for the review

| **Database** | **Search string** |
| --- | --- |
| PubMed | ((medical order entry systems[mh] OR medical order entry system*[tiab] OR computerized order entry[tiab] OR computerized prescriber order entry[tiab] OR computerized provider order entry[tiab] OR computerized physician order entry[tiab] OR electronic order entry[tiab] OR electronic prescribing[mh] OR electronic prescribing[tiab] OR cpoe[tiab] OR drug-therapy, computer assisted[mh] OR computer assisted drug therapy[tiab] OR decision support systems, clinical[mh] OR decision support system*[tiab] OR reminder system*[tiab] OR decision-making, computer assisted[mh] OR computer assisted decision making [tiab] OR diagnosis, computer assisted[mh] OR computer assisted diagnosis[tiab] OR therapy, computer assisted[mh] OR computer assisted therapy[tiab] OR expert systems[mh] OR expert system*[tiab] OR *CDS*[tiab])) AND ((health*[tiab] OR medical[tiab] OR clinic*[tiab] OR hospital[tiab])) AND ((acceptance[tiab] OR adoption[tiab] OR intention to use[tiab] OR implementation[tiab] OR integration[tiab] OR introduction[tiab] OR rollout[tiab])) |
| Web of Science | (TS=((“order entry system$” OR “computer* order entry” OR “computer* prescriber order entry” OR “computer* provider order entry” OR “computer* physician order entry” OR “electronic order entry” OR “electronic prescribing” OR CPOE OR “computer* drug-therapy” OR “decision support system$” OR “reminder system$” OR “computer* decision-making” OR “computer* diagnos*” OR “computer* therapy” OR “expert system$” OR CDS OR CDSS) AND (health* OR medical OR clinic* OR hospital) AND (acceptance OR adoption OR “intention to use” OR implementation OR integration OR introduction OR rollout)  AND LANGUAGE: (English) AND DOCUMENT TYPES: (Article)  Indexes=SCI-EXPANDED, SSCI |

Table S2. Characteristics of included studies.

| **Authors, year published, country** | **Study type and type of data** | **Time-frame** | **Clinician type** | **CDSS** | **Implementation phase** | **Target area of medical care** | **CDSS’ applied medical specialty** |
| --- | --- | --- | --- | --- | --- | --- | --- |
| Abdel-Qader et al., 2010, UK [109] | Quantitative | Cross-sectional | Pharmacists, physicians, medical leaders, N = 342 | System developed for this study. Clinical manager: ordering (referral, medication, pathology) support, prescribing support, allergy and drug interaction support | Sustainment | Follow-up management, planning & implementing treatment | Mixed specialties |
| Abejirinde et al., 2018, Ghana [119] | Mixed method (focus groups, observation, survey) | Cross-sectional | Physicians, health workers, N = 24 | System developed for this study. System supports instant informed diagnosis during antenatal care by enabling noninvasive point-of-care screening for preeclampsia, gestational diabetes, and anemia. | Exploration | Diagnosis | Gynecology & obstetrics/maternal/antenatal/perinatal/neonatal care |
| Abidi et al., 2018, Canada [125] | Qualitative (focus groups, usability testing & think aloud protocol) | Cross-sectional | Physicians, certified diabetes educators, N = 10 | System developed for this study. System offers diabetes clinical practice guideline-based recommendations and behavior change strategies. | Active implementation | Planning & implementing treatment | Endocrinology |
| Abramson et al., 2012, USA [80] | Qualitative (interviews, observation) | Longitudinal | Physicians, N = 19 | Existing system. Systems offers prescription support, incl. drug-dosing alerts, drug-drug interactions, contraindications, allergic reactions, duplications, and information on dispensed prescriptions and medical visits. | Active implementation | Cost reduction & improved patient convenience, planning & implementing treatment | Internal medicine |
| Agarwal et al., 2010, USA [87] | Qualitative (interviews, observation, focus groups) | Cross-sectional | Physicians, nurse practitioners, medical leaders, N = 46 | Existing system. System not specified; used for e-prescription support. | Sustainment | Cost reduction & improved patient convenience, planning & implementing treatment | Mixed specialties |
| Ahmad et al., 2023, China [139] | Quantitative | Cross-sectional | Physicians, nurse practitioners, chemists, laboratory technicians, N = 237 | Non-existing system. Intention adoption factors on CDSS during COVID-19 pandemic emergency. | Exploration | Preventive care, diagnosis, planning & implementing treatment, follow-up management, hospital, provider efficiency, cost reduction & improved patient convenience | Mixed specialties |
| Anderson et al., 2014, USA [140] | Qualitative (interviews, video evaluation, usability testing & think aloud protocol) | Cross-sectional | Physicians, members of Stroke Quality Improvement network, N = 22 | System developed for this study. System designed to improve the management of stroke risk factors. | Exploration | Preventive care | Primary care, neurology, rehabilitation medicine |
| Arts et al., 2018, Netherlands [141] | Mixed method (focus groups, survey) | Cross-sectional | Physicians, N = 30 | System developed for this study. System includes two guideline domains, one relating to care of older adults and the other to anticoagulant management in atrial fibrillation. | Exploration | Diagnosis, planning & implementing treatment | Primary care |
| **Authors, year published, country** | **Study type and type of data** | **Time-frame** | **Clinician type** | **CDSS** | **Implementation phase** | **Target area of medical care** | **CDSS’ applied medical specialty** |
| Ash et al., 2011, USA [3] | Qualitative (interviews, observation) | Cross-sectional | Physicians, nurses, medical assistants, nurse practitioners, physician assistants, medical leaders, N = 27 | Existing system. System not specified, different CDSSs used within EHR in ambulatory settings in various practices. | Adoption decision, implementation preparation, sustainment | Follow-up management, hospital, provider efficiency, planning & implementing treatment | Primary care |
| Ash et al., 2020, USA [142] | Qualitative | Cross-sectional | Physicians, nurses, pharmacists, medical leaders, N = 83 | System developed for this study. System not specified, different CDSSs used in five different sites with three different EHR systems. | Sustainment | Follow-up management | Primary care |
| Ballard et al., 2013, USA [143] | Quantitative | Cross-sectional | Physicians, N = 399 | Existing system. Clinical prediction rules for pediatric head trauma patients. | Exploration | Planning & implementing treatment | Emergency medicine |
| Berge et al., 2023, Norway [134] | Mixed method (actual use measurements data/log files, documents, interviews, survey) | Cross-sectional | Physicians, nurses, N = 52 | System developed for this study. Algorithm-based CDSS for identifying and classifying patient allergies through text mining of the patient narrative. | Active implementation | Diagnosis, preventive care | Mixed specialties |
| Buenestado et al., 2013, Spain [144] | Quantitative | Cross-sectional | Physicians, N = 8 | Existing system. System provides possibility to examine several sources of complementary information, i.e. medical terminology, active principles and pharmaceutical products, bibliography and evidence supporting the recommendations. | Adoption decision, implementation preparation | Planning & implementing treatment | Pediatrics |
| Carayon et al., 2011, USA [145] | Quantitative | Cross-sectional | Nurses, N = 282 | Existing system. Computerized provider order entry (CPOE) and electronic medication administration record (eMAR) system. | Sustainment | Planning & implementing treatment | Intensive care |
| Carland et al., 2021, Australia [105] | Qualitative | Cross-sectional | Prescribers in general, N = 17 | Existing system. System intended for medication dosing decision support. | Exploration | Planning & implementing treatment | Pharmacology |
| Catho et al., 2020, Switzerland & France [146] | Qualitative | Cross-sectional | Physicians, medical residents, N = 29 | Existing system. System supports prescription of antibiotics, contains local antimicrobial guidelines. | Exploration | Planning & implementing treatment | Mixed specialties |
| Charani et al, 2013, UK [123] | Mixed method (actual use measurement/log files, focus groups, observation, survey) | Longitudinal | Physicians, pharmacists, medical leaders, N = 168 | System developed for this study. Smartphone application with integrated antibiotic prescribing policies. | Adoption decision, implementation preparation | Planning & implementing treatment | Pharmacology |
| Chow et al., 2015, Singapore [82] | Mixed method (focus groups, survey) | Cross-sectional | Physicians, N = 265 | System developed for this study. System offers patient-specific antibiotic recommendations at point of prescribing, integrated with CPOE. | Sustainment | Planning & implementing treatment | Mixed specialties |
| **Authors, year published, country** | **Study type and type of data** | **Time-frame** | **Clinician type** | **CDSS** | **Implementation phase** | **Target area of medical care** | **CDSS’ applied medical specialty** |
| Chung et al., 2017, USA [136] | Qualitative (interviews, focus groups) | Cross-sectional | Physicians, nurse practitioners, medical leaders, N = 22 | Existing system. System delivers recommendations at the point-of-care through the existing medical record system. Offers alerts and advice about antibiotics prescribing. | Active implementation | Planning & implementing treatment | Pediatrics |
| Cracknell, 2020, UK [99] | Mixed method (focus groups, survey) | Longitudinal | Physicians, nurse practitioners, medical leaders, N = 35 (survey), N = 5 (focus groups) | Existing system. System facilitates the communication of a prescription or medicine order, aiding the choice, administration and supply of a medicine. | Exploration | Planning & implementing treatment | Oncology |
| Cranfield et al., 2015, UK [137] | Qualitative | Longitudinal | Physicians, nurse practitioners, medical leaders, N = 72 | Existing system. System not specified; computerized physician order entry (CPOE) and picture archiving and communication systems (PACS) | Active implementation | Hospital, provider efficiency | Mixed specialties |
| Cresswell et al., 2017, UK [91] | Qualitative (interviews, documents, observation) | Longitudinal | Physicians, nurse practitioners, medical leaders, N = 173 | Existing system. Six different CPOE and CDS systems; both stand-alone systems and integrated into hospital information systems. | Adoption decision, implementation preparation, sustainment | Planning & implementing treatment | Emergency medicine |
| Cresswell et al., 2019, UK [77] | Qualitative (interviews, observation) | Longitudinal | Physicians, pharmacists, medical leaders, N = 30 | Existing system. System not specified. Evaluation of national programme to build pilot Decision Support Platform | Active implementation | Preventive care | Mixed specialties |
| De Vries et al., 2013, Netherlands [124] | Quantitative | Cross-sectional | Cardiologists, heart failure nurses, N = 162 | Existing system. System not specified; different CDSSs used for treatment of heart failure patients. | Sustainment | Cost reduction & improved patient convenience, diagnosis, planning & implementing treatment, Preventive care | Cardiology |
| de Wattewille et al., 2020, Switzerland [71] | Qualitative | Cross-sectional | Physicians, nurse practitioners, N = 20 | System developed for this study. System assists caregivers in achieving optimized blood glucose control coupled to nutritional support. | Exploration | Planning & implementing treatment | Mixed specialties |
| Devine et al., 2010, USA [11] | Qualitative (focus groups, interviews) | Cross-sectional | Physicians, nurses, medical assistants, N = 70 | Various systems existing systems i.e. systems used for e-prescribing. | Sustainment | Cost reduction & improved patient convenience, follow-up management, planning & implementing treatment | Primary care |
| Elnahal et al., 2011, USA [147] | Quantitative | Cross-sectional | Hospitals, N = 1637 | Various systems with decision support functionalities, not specified. | Sustainment | Cost reduction & improved patient convenience, diagnosis, follow-up management, hospital, provider efficiency, planning & implementing treatment, Preventive care | Mixed specialties |
| **Authors, year published, country** | **Study type and type of data** | **Time-frame** | **Clinician type** | **CDSS** | **Implementation phase** | **Target area of medical care** | **CDSS’ applied medical specialty** |
| English et al., 2017, USA [148] | Quantitative | Cross-sectional | Clinical pharmacists, N = 25 | Existing system. System provides real-time surveillance of pharmaceutical therapies. | Sustainment | Planning & implementing treatment | Pharmacology |
| Esmaeilzadeh et al., 2015, Malaysia [5] | Quantitative | Cross-sectional | Physicians, N = 335 | Non-specific CDSS. | Exploration | Diagnosis, planning & implementing treatment | Mixed specialties |
| Feldstein et al., 2013, USA [72] | Qualitative | Cross-sectional | Physicians, medical assistants, medical leaders, N = 52 | Existing system. System not specified; CDSS integrated in EHR and intended to manage care individual patients and patient panels. | Sustainment | Follow-up management, hospital, provider efficiency, planning & implementing treatment, Preventive care | Primary care |
| Finley et al., 2018, USA [73] | Qualitative | Cross-sectional | Physicians, nurse practitioners, N = 26 | System developed for this study. System intended for opiod prescription drug monitoring. | Exploration | Planning & implementing treatment | Mixed specialties |
| Ford et al., 2021, UK [83] | Qualitative | Cross-sectional | Physicians, N = 11 | Existing system. AI-based CDSS to identify people with dementia. | Exploration | Diagnosis | Primary care |
| Fossum et al., 2011, Norway [93] | Mixed method (usability testing & think aloud protocol, observation, survey) | Cross-sectional | Nurse practitioners, N = 25 | System developed for this study. System not specified; decision support for nursing care planning | Sustainment | Planning & implementing treatment | Nursing homes |
| Frisinger & Papachristou, 2023, Sweden [149] | Qualitative | Cross-sectional | Physicians, medical leaders, N = 15 | Existing system. AI-based CDSS for detection of malignant melanoma. | Exploration | Diagnosis | Primary care |
| Garabedian et al., 2022, USA [150] | Mixed method (contextual inquiry sessions, group design sessions, think-aloud sessions) | Longitudinal | Physicians, nurse practitioners, physician assistants, N = 20 | System (alert CDSS) developed for this study. The CDSS in the form of a best practice advisory alert provides support for hypertensive treatment recommendations for patients with chronic kidney disease. | Exploration | Planning & implementing treatment | Primary care |
| Gezer et al., 2023, Australia [151] | Qualitative | Cross-sectional | Physicians, N = 9 | CDSS module for the management of sexually transmitted infections to be developed based on the study results. | Exploration | Diagnosis, follow-up management | Primary care, HIV/infectiology |
| Greenberg et al., 2021, USA [127] | Qualitative | Cross-sectional | Physicians, N = 28 | Existing system. System offers support to guide clinicians’ decisions regarding the need for ICU admission. | Adoption decision, implementation preparation, sustainment | Diagnosis | Mixed specialties |
| Grout et al., 2018, USA [152] | Quantitative | Longitudinal | Physicians, nurse practitioners, N = 352 | Existing system. System uses patient data to generate patient screening questionnaires. | Sustainment | Preventive care | Pediatrics |
| Hains et al., 2010, Australia [89] | Mixed method (focus groups, interviews, survey) | Longitudinal | Physicians, nurse practitioners, medical residents, N = 207 | System developed for this study. System provides detailed and extensive instructions on how to deliver optimized evidence-based chemotherapy treatments safely and appropriately. | Active implementation | Planning & implementing treatment | Mixed specialties |
| **Authors, year published, country** | **Study type and type of data** | **Time-frame** | **Clinician type** | **CDSS** | **Implementation phase** | **Target area of medical care** | **CDSS’ applied medical specialty** |
| Harry et al., 2019, USA [78] | Qualitative | Cross-sectional | Physicians, nurse practitioners, medical leaders, N = 28 | System developed for this study. System not specified; offers decision support for cardiovascular risk management, human papillomavirus [HPV] vaccination) and breast, cervical, colorectal, lung cancer prevention. | Exploration | Planning & implementing treatment, Preventive care | Mixed specialties |
| Helldén et al., 2015, Sweden [153] | Mixed method (actual use measurement data/log files, focus groups, survey) | Cross-sectional | Physicians, N = 7 | System developed for this study. System offers medication decision support (alerts, drug lists etc.) | Sustainment | Planning & implementing treatment | Primary care |
| Hinderer et al., 2017, Germany [154] | Quantitative | Cross-sectional | Physicians, N = 564 | System to be developed. System checks prescription with genomic testing to improve efficacy and mitigate side effects for numerous medications, and proposes different medication if indication is given. | Exploration | Planning & implementing treatment | Mixed specialties |
| Holden, 2010, USA [10] | Qualitative | Cross-sectional | Physicians, N = 20 | Existing system. System not specified | Active implementation, sustainment | Planning & implementing treatment | Primary care |
| Hor et al., 2010, Ireland [155] | Quantitative | Cross-sectional | Physicians, N = 262 | Clinical Decision Support within the e-Prescribing context (CDS-eP). | Exploration | Planning & implementing treatment | Primary care |
| Hsiao & Chen, 2015, Taiwan [156] | Quantitative | Cross-sectional | Physicians, N = 238 | Existing system. System not specified | Sustainment | Planning & implementing treatment, preventive care | Mixed specialties |
| Hsiao et al., 2013, Taiwan [157] | Quantitative | Cross-sectional | Nurse practitioners, N = 101 | Existing system. System not specified; provides decision support capabilities to healthcare professionals during patient pain management. | Sustainment | Planning & implementing treatment | Internal medicine |
| Hsu et al., 2015, Hong Kong [112] | Quantitative | Cross-sectional | Medical residents, N = 259 | Existing system. System not specified. | Sustainment | Cost reduction & improved patient convenience, follow-up management | Mixed specialties |
| Huguet et al. 2023, USA [158] | Mixed method (actual use measurement data/log files, interviews) | Cross-sectional | Physicians, nurse practitioners, midwifes, medical assistants, N = 331 | Existing system. CDSS supports documenting patient’s cervical cancer screening results and tracking of their receipt of appropriate follow-up care. | Sustainment | Planning & implementing treatment, follow-up management, | Oncology |
| Jansen-Kosterink et al., 2021, Netherlands [74] | Quantitative | Cross-sectional | Physicians, physical therapists, N = 98 | Existing system. System provides support for personalized and faster evidence-based management of neck and low back pain | Adoption decision/implementation preparation | Planning & implementing treatment, preventive care | Primary care |
| **Authors, year published, country** | **Study type and type of data** | **Time-frame** | **Clinician type** | **CDSS** | **Implementation phase** | **Target area of medical care** | **CDSS’ applied medical specialty** |
| Jeffery et al., 2017, USA [34] | Qualitative | Cross-sectional | Nurse practitioners, N = 20 | Both existing system and system developed for this study. System not specified | Exploration | Diagnosis | Cardiology |
| Jeffries et al., 2018, UK [92] | Qualitative | Cross-sectional | Physicians, pharmacists, general practice staff, medical leaders, N = 28 | Existing system. System intended for medication prescription support. | Active implementation | Diagnosis, preventive care | Mixed specialties |
| Jeffries et al., 2021, UK [159] | Qualitative | Longitudinal | Physicians, pharmacists, pharmacy technicians, nurse practitioners, N = 39 | Existing system. System intended for medication prescription support. | Sustainment | Cost reduction & improved patient convenience, preventive care | Primary care |
| Jeffries et al., 2023, UK [160] | Qualitative | Cross-sectional | Physicians, nurse practitioners, pharmacists, N = 23 | Existing system. System not specified. | Sustainment | Planning & implementing treatment, preventive care | Primary care |
| Jeng & Tzeng, 2012, Taiwan [161] | Quantitative | Cross-sectional | Physicians, N = 98 | System not specified. | Adoption decision/implementation preparation | Diagnosis | Mixed specialties |
| Jeon et al., 2014, Canada [162] | Mixed method (focus groups, survey) | Cross-sectional | Physicians, nurse practitioners, N = 24 | Existing system. Systems not specified; CPOEs used in oncology institutions. | Sustainment | Planning & implementing treatment | Oncology |
| Jung et al., 2020, South Korea [90] | Mixed method (interviews, survey) | Cross-sectional | Physicians, N= 61 | Existing system. System not specified; CDSS intended for medication decision support | Sustainment | Planning & implementing treatment | Mixed specialties |
| Kanagasundaram et al., 2015, UK [163] | Qualitative | Cross-sectional | Physicians, medical residents, N = 24 | Both existing system and system developed for this study. System not specified | Exploration | Planning & implementing treatment | Nephrology |
| Kastner et al., 2010, Canada [164] | Qualitative (focus groups) | Cross-sectional | Physicians, N = 61 | System developed for this study. CDSS for osteoporosis disease management | Exploration | Planning & implementing treatment | Mixed specialties |
| Khajouei et al., 2011, Netherlands [165] | Quantitative | Cross-sectional | Physicians, nurse practitioners, N = 433 | Existing system. System offers medication decision support (alerts, drug lists etc.) | Sustainment | Planning & implementing treatment | Mixed specialties |
| Khan et al., 2016, USA [166] | Qualitative (focus group, observation, interviews) | Cross-sectional | Physicians, N = 5 | Existing system. System not specified; offers pulmonary embolism clinical prediction rules. | Adoption decision/implementation preparation | Diagnosis, preventive care | Emergency medicine |
| Klarenbeek et al., 2021, Netherlands [104] | Qualitative | Cross-sectional | Physicians, N = 26 | System developed for this study. System not specified; CDSS intended for oncological treatment support. | Adoption decision/implementation preparation | Hospital, provider efficiency, planning & implementing treatment | Oncology |
| Kortteisto et al., 2012, Finland [113] | Mixed method (focus groups, survey) | Longitudinal | Physicians, nurse practitioners, N = 48 | Existing system. System not specified; offers drug alerts, reminders, guideline links, and virtual health checks | Sustainment | Planning & implementing treatment | Primary care |
| **Authors, year published, country** | **Study type and type of data** | **Time-frame** | **Clinician type** | **CDSS** | **Implementation phase** | **Target area of medical care** | **CDSS’ applied medical specialty** |
| Koskela et al., 2016, Finland [120] | Qualitative (focus groups) | Cross-sectional | Physicians, nurse practitioners, N = 21 | System developed for this study. System provides patient-specific clinical recommendations or warnings in the form of reminders and links to guidelines. | Active implementation | Preventive care | Mixed specialties |
| Laka et al. 2021, Australia [69] | Quantitative | Cross-sectional | Physicians, N = 180 | Existing system. System not specified; offers decision support for antibiotic medication treatment. | Sustainment | Planning & implementing treatment | Pharmacology |
| Langton et al., 2013, Australia [167] | Quantitative (Actual use measurement data / log files, survey) | Longitudinal | Physicians, N = 2549 | Both existing system and system developed for this study. System provides detailed and extensive instructions on how to deliver optimized evidence-based chemotherapy treatments safely and appropriately. | Sustainment | Cost reduction & improved patient convenience, follow-up management, Planning & implementing treatment | Mixed specialties |
| Liang et al., 2010, China [168] | Quantitative | Longitudinal | Physicians, N = 103 | Existing system. CPOE system for electronic prescriptions and lab orders. | Sustainment | planning & implementing treatment | Mixed specialties |
| Liberati et al., 2017, Italy [24] | Qualitative | Cross-sectional | Physicians, nurse practitioners, medical leaders, N = 30 | Existing system. System offers patient-specific, point-of-care reminders. Advice on diagnosis or medication or laboratory test decisions, integrated in EHR. | Exploration, adoption decision/implementation preparation, active implementation, sustainment | Diagnosis, follow-up management | Oncology, orthopedics |
| Litvin et al., 2012, USA [169] | Mixed method (actual use measurement/log files, interviews) | Cross-sectional | Physicians, nurse practitioners, physician assistants, N = 39 | System developed for this study. System offers decision support for diagnosis, scoring strategies, and appropriate antibiotic use. | Exploration | Planning & implementing treatment | Primary care |
| Liu et al., 2022, USA [170] | Quantitative | Cross-sectional | Physicians, nurse practitioners, physician assistants, N = 116 | Existing system. System provides order sets for pain management. | Sustainment | Planning & implementing treatment | Mixed specialties |
| Liu et al., 2023, USA [84] | Qualitative | Cross-sectional | Pharmacists, N = 13 | System developed for this study. AI-based CDSS that provides medication support (dose and interval). | Sustainment | Preventive care | Intensive care |
| Lugtenberg et al., 2015, Netherlands [171] | Quantitative | Cross-sectional | Physicians, nurse practitioners, N = 164 | Existing system. System offers patient-specific advices during consultation in terms of patient data registration, drug prescription and management. | Active implementation | Planning & implementing treatment | Primary care |
| Lugtenberg et al., 2015, Netherlands [79] | Qualitative (focus groups) | Cross-sectional | Physicians, nurse practitioners, medical residents, N = 24 | Existing system. System offers patient-specific advices during consultation in terms of patient data registration, drug prescription and management. | Active implementation | Planning & implementing treatment | Primary care |
| **Authors, year published, country** | **Study type and type of data** | **Time-frame** | **Clinician type** | **CDSS** | **Implementation phase** | **Target area of medical care** | **CDSS’ applied medical specialty** |
| Malo et al., 2012, Canada [172] | Quantitative | Cross-sectional | Nurse practitioners, N = 87 | System developed for this study. System intended for charting of administered medication, intravenous line use, and general information related to patient care. Offers clinical action alerts. | Exploration | Hospital, provider efficiency, planning & implementing treatment | Emergency medicine |
| Maslej et al., 2023, Canada [173] | Vignette experiment in laboratory setting | Cross-sectional | Physicians, psychiatrists, residents, N = 83 | Existing system. AI-based CDSS providing clinical note summary and treatment recommendation for patients with major depressive disorder. | Exploration | Planning & implementing treatment | Primary care, psychiatry |
| Masterson Creber et al., 2018, USA [70] | Mixed method (documents, interviews) | Cross-sectional | Physicians, nurse practitioners, medical residents, medical leaders, N = 38 | System developed for this study. The clinical decision support rules provide risk estimates to decrease use of CTs in children with minor blunt head trauma. | Exploration | Preventive care | Pediatrics |
| McDermott et al., 2010, UK [115] | Qualitative | Cross-sectional | Physicians, N = 33 | System developed for this study. CDSS for antibiotic prescribing | Exploration | Hospital, provider efficiency, planning & implementing treatment | Primary care |
| McParland et al., 2019, UK [30] | Qualitative (focus group) | Cross-sectional | Physicians, N = 29 | Existing system. Differential Diagnosis Decision Support System (DDDSS) provides support in determining differential diagnosis. | Exploration | Diagnosis | Primary care |
| Mertz et al., 2017, USA [94] | Quantitative | Longitudinal | Dentists, dental specialists, N = 508 | Existing system. Systems offers caries management, periodontal disease management and risk assessment-based proactive dental care. | Sustainment | Planning & implementing treatment | Dental care |
| Meulendijk et al., 2013, Netherlands [174] | Quantitative | Cross-sectional | Physicians, N = 184 | System developed for this study. System intended for medication prescription support. | Sustainment | Diagnosis, follow-up management, Planning & implementing treatment | Primary care |
| Mozaffar et al., 2016, UK [175] | Qualitative | Cross-sectional | Clinical staff, N = 214 | Existing system. CPOE and CDSS for prescribing | Adoption decision/implementation preparation, active implementation, sustainment | Planning & implementing treatment | Emergency medicine |
| Mozaffar et al., 2017, UK [111] | Qualitative | Longitudinal | Clinical staff, N = 214 | Existing system. CPOE and CDSS for prescribing | Sustainment | Planning & implementing treatment | Emergency medicine |
| Noormohammad et al., 2010, Kenya [110] | Mixed method (documents, interviews, observation) | Cross-sectional | Nurse practitioners, N not specified (data from 1,300 clinical cases) | System newly developed and implemented in one clinic in Kenya for this study. CDSS for care of HIV-patients | Active implementation | Preventive care | HIV/infectiology |
| Overby et al., 2014, USA [108] | Quantitative | Cross-sectional | Physicians, N = 101 | System developed for this study. CDSS focuses on presenting genetic variants with established clinical significance. | Exploration | Planning & implementing treatment | Internal medicine |
| **Authors, year published, country** | **Study type and type of data** | **Time-frame** | **Clinician type** | **CDSS** | **Implementation phase** | **Target area of medical care** | **CDSS’ applied medical specialty** |
| O’Sullivan et al., 2014, Canada [114] | Quantitative | Cross-sectional | Physicians, N = 39 | Existing system. CDSS to help with the management of pediatric asthma exacerbation in the Emergency Department | Exploration, adoption decision/implementation preparation | Diagnosis, follow-up management, Planning & implementing treatment, Preventive care | Emergency medicine |
| Patel et al., 2015, UK [126] | Mixed method (actual use measurement/log files, interviews) | Longitudinal | Physicians, N = 16 | Existing system. System not specified | Exploration | Planning & implementing treatment | Internal medicine |
| Paulsen et al., 2019, Norway [176] | Qualitative (interviews, focus groups) | Cross-sectional | Physicians, nurse practitioners, registered dietitians, medical leaders, N = 27 | System developed for this study. System offers decision support to prevent and treat disease-related malnutrition. | Exploration | Planning & implementing treatment | Nutritional care |
| Peute et al., 2010, Netherlands [103] | Mixed method (documents, interviews, observation, survey) | Longitudinal | Physicians, medical leaders, N = 11 | Existing system. System intended for laboratory ordering | Exploration | Hospital, provider efficiency | Neurology |
| Pevnick et al., 2010, USA [177] | Quantitative (actual use measurement data/log files, documents) | Longitudinal | Physicians, N = 297 | Existing system. System used for e-prescriptions.. | Adoption decision/implementation preparation | Planning & implementing treatment | Mixed specialties |
| Ploegmakers et al., 2022, Austria, Belgium, Czech Republic, Denmark, Finland, Italy, The Netherlands, Poland, Spain, Turkey, UK [68] | Quantitative | Cross-sectional | Physicians, N = 581 | CDSS not specified. | Sustainment | Preventive care | Geriatrics |
| Porter et al., 2018, UK [102] | Qualitative (interviews, focus groups) | Longitudinal | Paramedics, N = 20 | Existing system. System assists paramedics in referring patients to community-based care. | Exploration | Planning & implementing treatment | Emergency medicine |
| Pratt et al., 2022, USA [178] | Qualitative | Cross-sectional | Physicians nurse practitioners, clinical assistants, N = 24 | Existing system. CDSS designed to identify and provide care recommendations for adults with prediabetes. | Adoption decision/implementation preparation | Preventive care | Primary care |
| Randell & Dowding, 2010, UK [98] | Qualitative (interviews, documents, clinical trial data, observation) | Longitudinal | Nurse practitioners, medical leaders, N = 124 | Four different systems with decision support functionalities. | Sustainment | Diagnosis, hospital, provider efficiency, Planning & implementing treatment | Mixed specialties |
| **Authors, year published, country** | **Study type and type of data** | **Time-frame** | **Clinician type** | **CDSS** | **Implementation phase** | **Target area of medical care** | **CDSS’ applied medical specialty** |
| Richardson & Ash, 2011, USA [35] | Qualitative (interviews, observation) | Cross-sectional | Physicians, N = 30 | Existing system. Various systems with decision support functionalities, not specified. | Active implementation | Cost reduction & improved patient convenience, follow-up management, hospital, provider efficiency, Planning & implementing treatment, Preventive care | Mixed specialties |
| Rieckert et al., 2018, Germany [122] | Qualitative | Cross-sectional | Physicians, N = 21 | System developed for this study. The clinical decision support tool reduces inappropriate medication in their older polypharmacy patients. | Exploration | Planning & implementing treatment | Primary care |
| Robertson et al., 2011, Australia [179] | Qualitative | Cross-sectional | Physicians, medical residents, N = 27 | Existing system. Various systems with decision support functionalities; not specified. | Sustainment | Follow-up management, Planning & implementing treatment, Preventive care | Primary care |
| Rock et al., 2022, USA [180] | Mixed method (actual test data, inquiries, focus groups, interviews) | Longitudinal | Physicians, residents, nurse practitioners, physician assistants, infection prevention practitioners, N = 47 | Existing system. CDSS provides support for identifying patients who should be tested for an infection. | Sustainment | Diagnosis | HIV/infectiology |
| Roebroek et al., 2020, Netherlands [181] | Qualitative | Cross-sectional | Psychiatrists, nurse practitioners, N = 13 | System developed for this study. CDSS combines routine outcome monitoring data with current treatment guidelines and care standards to provide clinicians and patients with personalized evidence-based treatment recommendations. | Exploration | Planning & implementing treatment | Psychiatry |
| Russ et al., 2012, USA [75] | Qualitative (interviews, observation, actual use measurement data / log files) | Cross-sectional | Physicians, clinical pharmacists, nurse practitioners, N = 320 | Existing system. CDSS for medication prescriptions | Sustainment | Preventive care | Primary care |
| Salwei et al., 2021, USA [182] | Qualitative | Cross-sectional | Physicians, N = 12 | Existing system. CDSS provides risk scoring algorithms for pulmonary embolism. | Active implementation | Diagnosis, preventive care | Emergency medicine |
| Sambasivan et al., 2012, Malaysia [48] | Quantitative | Cross-sectional | Physicians, N = 309 | Existing system. CDSS | Sustainment | Not specified | Mixed specialties |
| Sayood et al., 2021, USA [183] | Qualitative | Cross-sectional | Pharmacists, N = 21 | Hypothetical CDSS to assist in the management of common patient questions. | Adoption decision/implementation preparation | Preventive care | HIV / infectiology |
| Sedlmayr et al., 2013, Germany [107] | Mixed method (survey, observation) | Cross-sectional | Physicians, nurse practitioners, pharmacists, N = 9 | System developed for this study. System intended for medication support. | Active implementation | Planning & implementing treatment | Emergency medicine |
| **Authors, year published, country** | **Study type and type of data** | **Time-frame** | **Clinician type** | **CDSS** | **Implementation phase** | **Target area of medical care** | **CDSS’ applied medical specialty** |
| Seliaman & Albahly, 2023, Saudi Arabia [184] | Quantitative | Cross-sectional | Physicians, pharmacists, N = 116 | Existing system. CDSS provides various support, i.e., pharmacy, laboratory information system, infection control, home health care, rehabilitation etc. | Sustainment | Diagnosis, planning & implementing treatment, hospital & provider efficiency | Mixed specialties |
| Sheehan et al., 2013, USA [185] | Qualitative (interviews, observation, focus groups) | Cross-sectional | Medical leaders, N = 126 | Existing system. The clinical decision support rules provide risk estimates to decrease use of CTs in children with minor blunt head trauma. | Exploration | Planning & implementing treatment | Pediatrics |
| Shi et al., 2021, USA [186] | Quantitative | Longitudinal | Clinics and medical groups, N = 821 | Existing systems. Seven different CDSSs, not specified. | Sustainment | Preventive care, planning & implementing treatment | Mixed specialties |
| Shibl et al., 2013, Australia [12] | Qualitative | Cross-sectional | Physicians, N = 37 | Various systems with decision support functionalities; not specified. | Adoption decision/implementation preparation | Planning & implementing treatment | Primary care |
| Sicotte et al., 2013, Canada [187] | Mixed method (actual use measurement/log files, survey) | Cross-sectional | Physicians, N = 370 | Both existing system and system developed for this study. Systems offers prescription support, incl. drug-dosing alerts, drug-drug interactions, contraindications, allergic reactions, duplications, and information on dispensed prescriptions and medical visits. | Exploration | Cost reduction & improved patient convenience, Planning & implementing treatment, Preventive care | Primary care |
| Simon et al., 2013, USA [88] | Qualitative | Cross-sectional | Physicians, N = 24 | Existing system. System not specified. | Adoption decision/implementation preparation, sustainment | Hospital, provider efficiency | Mixed specialties |
| Singh et al., 2011, USA [86] | Quantitative | Cross-sectional | Physicians, clinical staff, N = 224 | Existing system. System not specified. | Exploration | Follow-up management, hospital, provider efficiency | Mixed specialties |
| Singh et al., 2018, India & Pakistan [9] | Mixed method (clinical trial data, interviews) | Longitudinal | Physicians, N = 558 | System developed for this study. System used for management of diabetic care goals | Active implementation, sustainment | Preventive care | Endocrinology |
| Snyder et al., 2021, USA [188] | Mixed method (observation, actual use measurement data/log files, interviews) | Cross-sectional | Pharmacists, N = 9 | Existing system. CDSS offers decision support for medication therapy management | Sustainment | Planning & implementing treatment | Pharmacy |
| Söling et al., 2023, Germany, [17] | Mixed method (interviews, focus groups, survey) | Cross-sectional | Physicians, N = 27 (interviews); Physicians, N = 179 (survey) | Existing system. System intended for medication and prescription support. | Sustainment | Diagnosis, preventive care, planning & implementing treatment | Primary care |
| Sukums et al., 2015, Ghana & Tanzania [97] | Mixed method (interviews, observation, survey) | Cross-sectional | Maternal and child health workers, N = 117 | System developed for this study. System provides decision support during antenatal and intrapartum care. | Adoption decision/implementation preparation | Preventive care | Gynecology & obstetrics/maternal/antenatal/perinatal/neonatal care |
| Tabla et al., 2022, France [189] | Quantitative | Cross-sectional | Physicians, N = 200 | Hypothetical AI-based CDSS with a diagnostic or therapeutic decision support module | Exploration, adoption decision/implementation preparation | Planning & implementing treatment, diagnosis | Primary care |
| **Authors, year published, country** | **Study type and type of data** | **Time-frame** | **Clinician type** | **CDSS** | **Implementation phase** | **Target area of medical care** | **CDSS’ applied medical specialty** |
| Teferi et al., 2022, Ethopia [190] | Quantitative | Cross-sectional | Physicians, residents, dentists, other clinical specialists, N = 384 | Existing system. CDSS offers decision support e-prescription. | Exploration | Preventive care | Primary care |
| Thomas et al., 2012, USA [191] | Quantitative | Cross-sectional | Prescribers, N = 246 | Existing system. System not specified. | Adoption decision/implementation preparation | Planning & implementing treatment | Mixed specialties |
| Trafton et., 2010, USA [130] | Mixed method (actual use measurement/log files, interviews, observation) | Longitudinal | Psychiatrists, Nurse practitioners, N = 9 | System developed for this study. CDSS provides recommendations for pain management | Adoption decision/implementation preparation | Cost reduction & improved patient convenience, diagnosis, Planning & implementing treatment | Primary care, psychiatry |
| Usmanova et al., 2020, India [8] | Qualitative | Cross-sectional | Nurse practitioners, medical leaders, N = 44 | System developed for this study. System supports providers’ management of mothers and neonates during the peripartum period. | Sustainment | Preventive care | Gynecology & obstetrics/maternal/antenatal/perinatal/neonatal care |
| Van Biesen et al., 2022, Belgium [192] | Mixed method (laboratory vignette study with thinking aloud approach) | Cross-sectional | Physicians, N = 30 | Hypothetical system used in study, based on existing system. Hypothetical CDSS provides support for medication treatment orders, and diagnosis. | Exploration | Diagnosis, planning & implementing treatment | Mixed specialties |
| Van Cauwenberge et al., 2022, Belgium [193] | Mixed method (clinical case vignettes, think-aloud protocol, interviews) | Cross-sectional | Physicians, N = 30 | Hypothetical system used in study, based on existing system. Hypothetical CDSS provides support for medication treatment orders, and diagnosis. | Exploration | Diagnosis, planning & implementing treatment | Mixed specialties |
| Vandenberg et al., 2017, USA [194] | Qualitative | Longitudinal | Physicians, medical residents, N = 20 | System developed for this study. System not specified; geriatric order sets | Sustainment | Hospital, provider efficiency | Emergency medicine, geriatrics |
| Varsi et al., 2023, Norway [195] | Qualitative | Longitudinal | Nurse practitioners, N = 12 | Existing system. CDSS is a digital dietary assessment and decision-support system designed to prevent and treat disease-related malnutrition. | Active implementation | Planning & implementing treatment | Geriatrics |
| Wannheden et al., 2017, Sweden [118] | Qualitative (focus groups) | Cross-sectional | Physicians, nurse practitioners, N = 14 | System developed for this study. System prototype for evidence-based drug therapy recommendations for HIV/TB treatment. | Exploration | Planning & implementing treatment | Geriatrics |
| Westerbeek et al., 2022, Netherlands [196] | Qualitative (focus groups) | Cross-sectional | Physicians, N = 13 | Existing system. CDSS offers decision support for medication-related falls. | Active implementation | Preventive care | Primary care |
| Wickström et al. 2020, Sweden [116] | Qualitative | Cross-sectional | Physicians, nurse practitioners, assistant nurses, N = 11 | System for clinical assessment of hard-to-heal ulcers | Exploration | Diagnosis, planning & implementing treatment | Wound management |
| Wijnhoven, 2022, country not specified [197] | Qualitative | Cross-sectional | Physicians, medical leaders, N = 23 | Existing system. CDSS for deciding on administering antibiotics to prematurely born babies. | Exploration | Planning & implementing treatment | Pediatrics |
| **Authors, year published, country** | **Study type and type of data** | **Time-frame** | **Clinician type** | **CDSS** | **Implementation phase** | **Target area of medical care** | **CDSS’ applied medical specialty** |
| Wrzosek et al., 2020, Poland [106] | Quantitative | Cross-sectional | Physicians, N = 144 | System developed for this study. System offers decision support e-prescription. | Adoption decision/implementation preparation | Planning & implementing treatment | Pharmacology |
| Yui et al., 2012, Taiwan [198] | Quantitative | Cross-sectional | Physicians, N = 225 | Existing system. System not specified; CPOE offers functions such as physician order entry, basic patient information inquiry, and medical records entry, etc. | Active implementation | Planning & implementing treatment | Mixed specialties |
| Zaidi & Marriott, 2012, Australia [85] | Quantitative | Cross-sectional | Physicians, pharmacists, medical residents, N = 115 | Existing system. CDSS for antibiotic medication treatment. | Active implementation | Planning & implementing treatment | Pharmacology |
| Zakane et al., 2014, Burkina Faso [199] | Qualitative | Cross-sectional | Nurse practitioners, N = 45 | System developed for this study. System based on WHO guidelines for maternal and neonatal care. | Adoption decision/implementation preparation | Planning & implementing treatment, Preventive care | Gynecology & obstetrics/maternal/antenatal/perinatal/neonatal care |
| Zha et al., 2022, China [200] | Quantitative | Cross-sectional | Nurse practitioners, N = 1100 | Existing system. AI-based CDSS for risk stratification of cardiovascular events.. | Sustainment | Preventive care | Cardiology |
| Zhai et al., 2022, China [121] | Mixed method (interviews, survey, observation) | Longitudinal | Nurse practitioners, N = 324 survey respondents, 20 interviewees | Existing system. CDSS intended to support nurse practitioners, contains evidence-based nursing knowledge base and big data analysis resources. | Sustainment | Diagnosis, planning & implementing treatment | Mixed specialties |
| Zhai et al., 2022, China [201] | Qualitative (interviews, observation) | Longitudinal | Nurse practitioners, N = 21 | Existing system. CDSS intended to support nurse practitioners, contains evidence-based nursing knowledge base and big data analysis resources. | Sustainment | Diagnosis, planning & implementing treatment | Mixed specialties |

Unless otherwise noted, the qualitative-only studies involved interviews and the quantitative-only studies involved sur
